# Self-driving laboratory platform for many-objective self-optimisation of polymer nanoparticle synthesis with cloud-integrated machine learning and orthogonal online analytics†

**DOI:** 10.1039/d5py00123d

**Published:** 2025-02-11

**Authors:** Stephen T. Knox, Kai E. Wu, Nazrul Islam, Roisin O'Connell, Peter M. Pittaway, Kudakwashe E. Chingono, John Oyekan, George Panoutsos, Thomas W. Chamberlain, Richard A. Bourne, Nicholas J. Warren

**Affiliations:** a School of Chemical and Process Engineering, University of Leeds Woodhouse Lane Leeds West Yorkshire UK nicholas.warren@sheffield.ac.uk; b School of Electrical and Electronic Engineering, Sir Frederick Mappin Building, The University of Sheffield Mappin Street Sheffield S1 4DT UK; c Department of Computer Science, University of York Deramore Lane York YO10 5GH UK; d School of Chemistry, University of Leeds Woodhouse Lane Leeds West Yorkshire UK; e School of Chemical, Materials and Biological Engineering, University of Sheffield Sir Robert Hadfield Building Mappin Street Sheffield S1 3JD UK

## Abstract

The application of artificial intelligence and machine learning is revolutionising the chemical industry, with the ability to automate and self-optimise reactions facilitating a step change in capability. Unlike small-molecules, polymer nanoparticles require navigation of a more complex parameter space to access the desired performance. In addition to the chemical reaction, it is desirable to optimise the polymer molecular weight distribution, particle size and polydispersity index. To solve this *many-objective* optimisation problem, a self-driving laboratory is constructed which synthesises and characterises polymer nanoparticles (incorporating NMR spectroscopy, gel permeation chromatography and dynamic light scattering). This facilitates the autonomous exploration of parameter space with programmable screens or AI driven optimisation campaigns *via* a cloud-based framework. The RAFT polymerisation of diacetone acrylamide mediated by a poly(dimethylacrylamide) macro-CTA was optimised to maximise monomer conversion, minimise molar mass dispersity, and target 80 nm particles with minimised polydispersity index. A full-factorial screen between 6- and 30-minutes residence time, between 68 and 80 °C and between 100 and 600 for the [monomer] : [CTA] ratio enabled mapping of the reaction space. This facilitated *in-silico* simulations using a range of algorithms – Thompson sampling efficient multi-objective optimisation (TSEMO), radial basis function neural network/reference vector evolutionary algorithm (RBFNN/RVEA) and multi objective particle swarm optimisation, hybridised with an evolutionary algorithm (EA-MOPSO), which were then applied to in-lab optimisations. This approach accounts for an unprecedented number of objectives for closed-loop optimisation of a synthetic polymerisation; and enabled the use of algorithms operated from different geographical locations to the reactor platform.

## Abstract 2

A self-driving laboratory, combining automated synthesis, characterisation, and cloud-based AI, was developed to optimise the synthesis of polymer nanoparticles by RAFT dispersion polymerisation.

## Graphical abstract

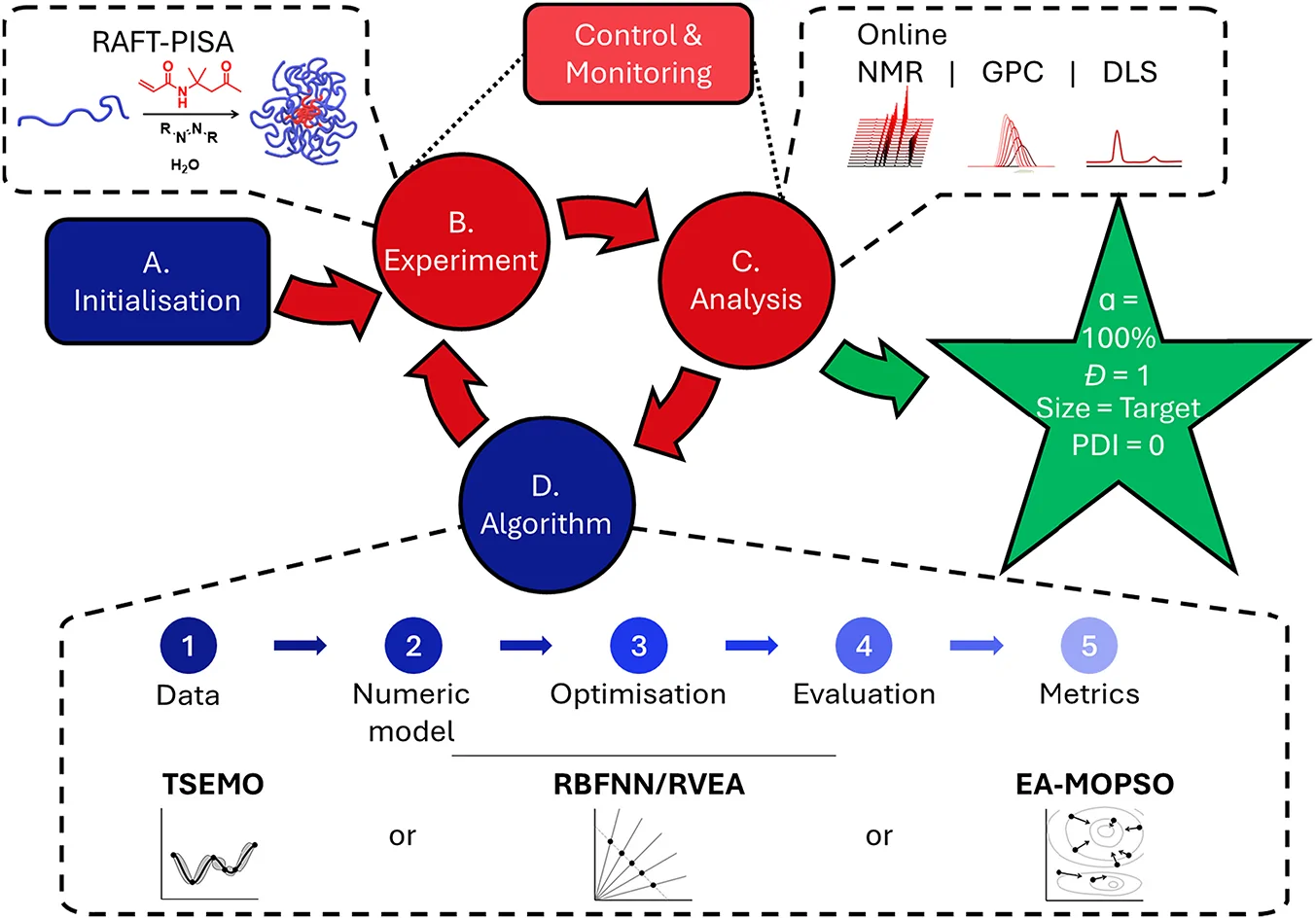

## Introduction

Optimising chemical processes is by no means a trivial endeavour, with complex responses to changes in inputs and a whole host of possible (often competing) objectives. However, with the rapid expansion of the capability in automated synthesis and analysis, coupled with integrated machine learning algorithms, there are a wide range of opportunities for innovation.^1–5^ Automation increases the quality of data, frees researchers from arduous and time-consuming tasks, and can identify optima either missed entirely by humans or increase efficiency in achieving such optima.^6^

Where automated synthesis, analysis and experimental selection can be operated without the need for human intervention, in a so-called “closed-loop” fashion, the impact of such self-driving laboratories can be dramatic and extensive. While there are a range of effective demonstrations for small molecule chemistry, the landscape for polymer chemistry is sparser, especially for more complex problems with greater than two objectives.^7,8^ That said, there are some examples demonstrating the combination of automated experimentation and machine learning algorithms. Single objective closed-loop optimisation has been demonstrated by Junkers and co-workers for both molecular weight^9^ and monomer conversion,^10^ allowing the targeting of a singular property of the polymer. Multi-objective problems offer the additional complexity that instead of a single optimum there are usually a set of non-dominated optima, where objectives trade-off against one another. The generation of such a “Pareto front” requires more sophistication in terms of algorithm and often a greater number of iterations of experiment to achieve success.^11^

Whilst Houben *et al.* demonstrated the manual multi-objective AI-assisted optimisation of an emulsion polymerisation formulation,^12^ significantly more automation was introduced by Leibfarth and co-workers, in a human-in-the-loop approach to optimising RAFT polymerisation.^13^ Fully closed-loop multi-objective optimisation was first demonstrated by Warren and co-workers, elucidating the trade-off between monomer conversion (*α*) and molecular weight dispersity (*Đ*) for a range of RAFT polymerisations.^14^

Additional complexity in polymer materials is introduced by considering that chains are often comprised of multiple, chemically different blocks, which each impart unique properties in solution due to spontaneous assembly into nanoparticles. As a result, their performance not only relies upon the chemical structure of the individual polymer chains, but the size and morphology of the particles. Aqueous polymerisation induced self-assembly (PISA) is a highly precise and rational method of controlling *both* the dimensions of the polymer chains and the nanoparticle size and morphology.^15^ Several PISA formulations have been conducted in flow, and a particularly attractive, widely studied formulation is based on the block copolymer polydimethylacrylamide-poly(diacetone acrylamide) (PDMAm-PDAAm).^16–20^ This all-acrylamide system facilitates an “ultrafast” approach to the polymer synthesis, reducing reaction times to the order of 10 minutes. Furthermore, the power of online analysis has previously been exemplified for this system whereby benchtop NMR was able to obtain high resolution kinetic data;^18^ and Guild *et al.* used online small angle X-ray scattering (SAXS) to monitor the evolution of particle size.^21^ In the case of the latter technique, access to such (typically facility based) instruments is limited and expensive, and automated data processing requires complex workflows within often access limited software interfaces. As such, SAXS currently offers limited utility for closed-loop optimisation. On the contrary, while offering less comprehensive information (especially for more complex morphologies), dynamic light scattering (DLS) provides a much more convenient and accessible method of characterising particles – with automated data processing, and at a significantly more affordable cost. DLS has been demonstrated in flow for a range of systems, either by accounting for motion of particles during the measurement^22–27^ or through a stopped-flow approach,^28^ including notably in a self-driving laboratory platform for size targeting of polymer particles, applying a single objective optimisation algorithm.^29^

In bringing together analyses that characterise the polymerisation, molecular weight distribution and particle properties, an unprecedented number of objectives emerge for closed-loop optimisation of polymer particle synthesis (*i.e.* Monomer conversion, molar mass averages, molar mass dispersity, particle size, particle size polydispersity index, DLS count rate. This is increased further by considering calculable objectives such as economic cost, environmental metrics such as *E*-factor.). This increase in problem complexity requires careful consideration from an algorithmic perspective, resulting in the need to evaluate a range of potential machine learning algorithms. Collaboration with experts in machine learning and artificial intelligence is essential and can be facilitated by a cloud-based framework.^30^

**Fig. 1:**
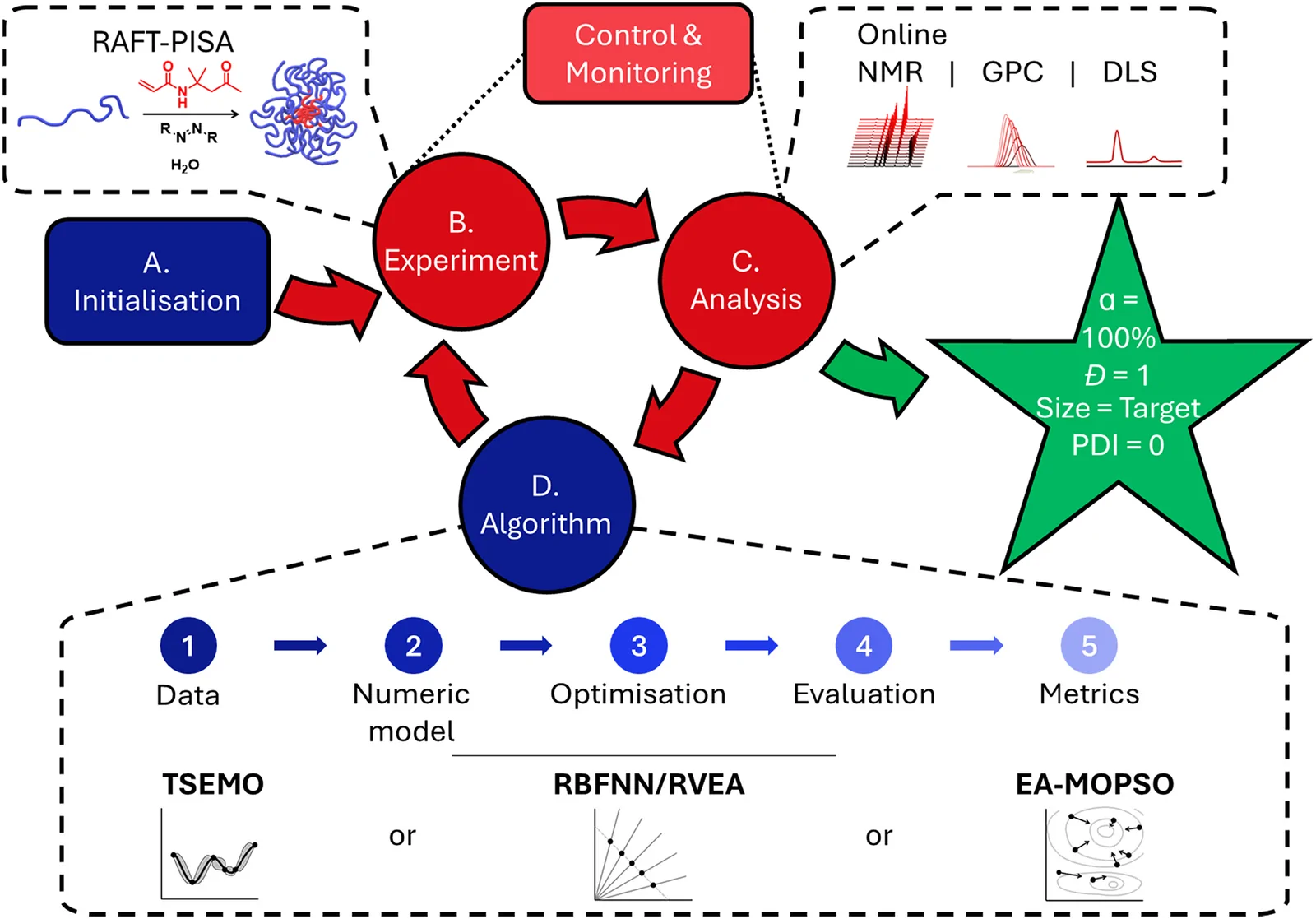
Generalised structure of an optimisation experiment, with reference to specific features applied in this work (as found in dashed boxes).

The structure of an optimisation experiment relying upon a machine learning algorithmic foundation (Fig. 1) is as follows: (A) the inputs and limits of those inputs for the system are established, and initialisation experiments selected within these limits (usually based upon a framework (*e.g.* Latin Hypercube Sampling (LHS), Design of Experiments (DoE)). (B) The selected experiments are performed, followed by (C) analysis of those experiments, to find the values for the objectives selected for the experiment. (D) The input variable-objective pairs are given to the algorithm which gives a new set of experimental conditions. Steps (B)–(D) are then repeated in the so-called closed-loop until certain criteria are fulfilled or user intervention halts the process. There exists a wide landscape of possible algorithms, with varying performance when applied to different chemical optimisation problems.^31,32^ In this work, a range of multi-objective optimisation algorithms were investigated to give diversity of behaviour, with Thompson sampling efficient multi-objective optimisation (TSEMO),^11^ radial basis function neural network/reference vector evolutionary algorithm (RBFNN/RVEA) and multi objective particle swarm optimisation, hybridised with an evolutionary algorithm (EA-MOPSO)^33^). The algorithms themselves operate with multiple steps (Fig. 1), beginning with (1) the hitherto obtained data, (2) which are then used to construct a surrogate model. (3) This can then be called by the optimisation algorithm to identify the location of the predicted Pareto front, and (4) an experiment selected using an evaluative methodology from these candidates. Finally, (5) the success of the optimisation process can be measured by a range of metrics, such as hypervolume (HV).^34^

Herein, the implementation of a platform to perform autonomous *many*-objective self-optimisation for particle synthesis *via* PISA, using a range of cloud-based machine learning algorithms is presented.

## Results and discussion

**Fig. 2:**
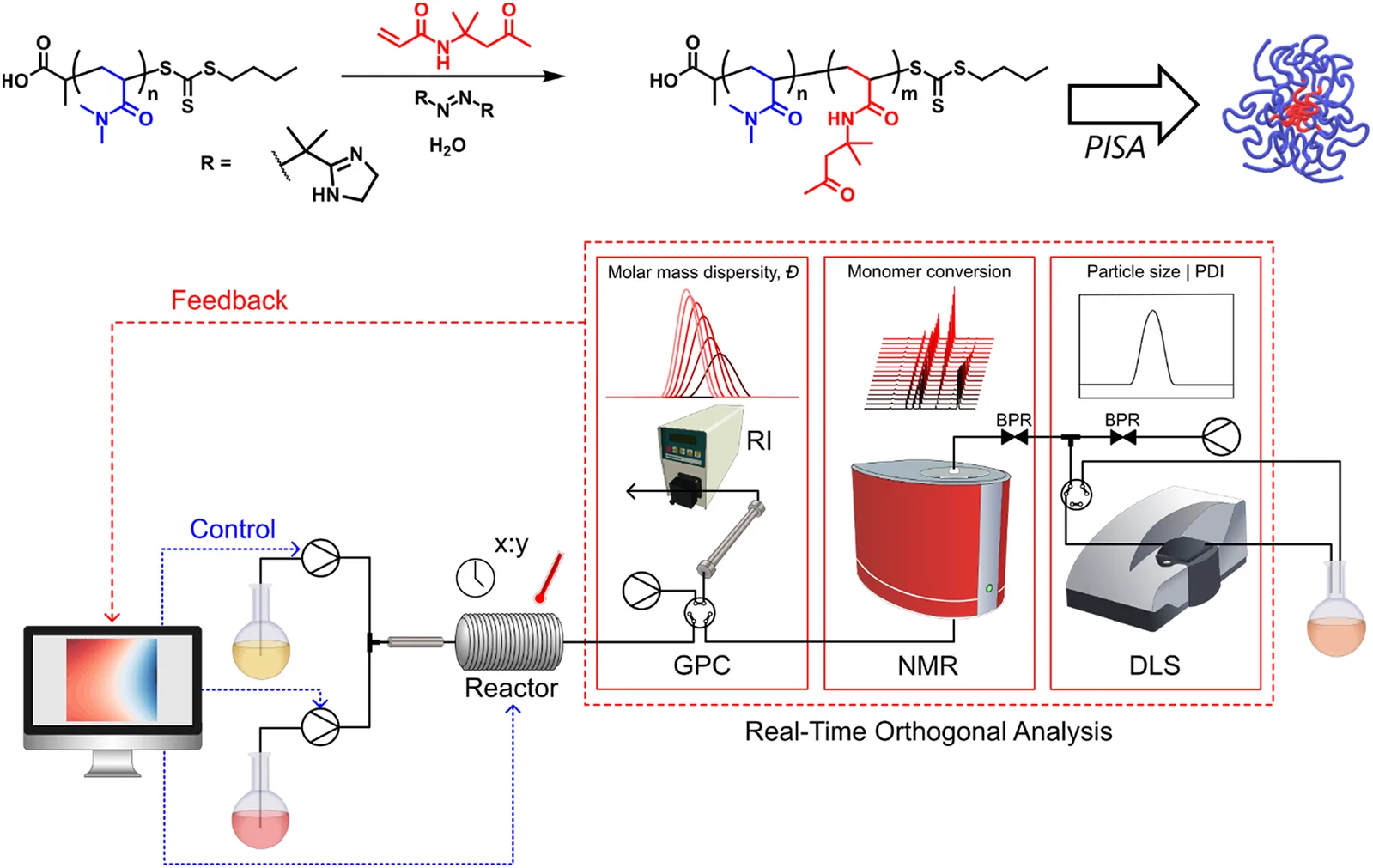
Overview of the chemistry and autonomous platform integrating a flow reactor and online gel permeation chromatography (GPC), ^1^H nuclear magnetic resonance (NMR) spectroscopy and dynamic light scattering (DLS) used in this work.

The platform used in this work follows from previous work^14^ and consists of (in brief) a tubular flow reactor, at-line gel permeation chromatography (GPC), inline benchtop nuclear magnetic resonance (NMR) spectroscopy and at-line dynamic light scattering (DLS) (Fig. 2; for a fuller description, see Fig. S1, in the ESI†). Of note is the volume of data available for each experiment using this platform – with monomer conversion from NMR spectroscopy, molecular weight information (number/weight average molecular weights, dispersity) from GPC and particle size information (average size, polydispersity index (PDI), count rate) from DLS, which gives unparalleled online information for a *closed-loop* autonomous polymerisation platform.

### High throughput screening

**Fig. 3:**
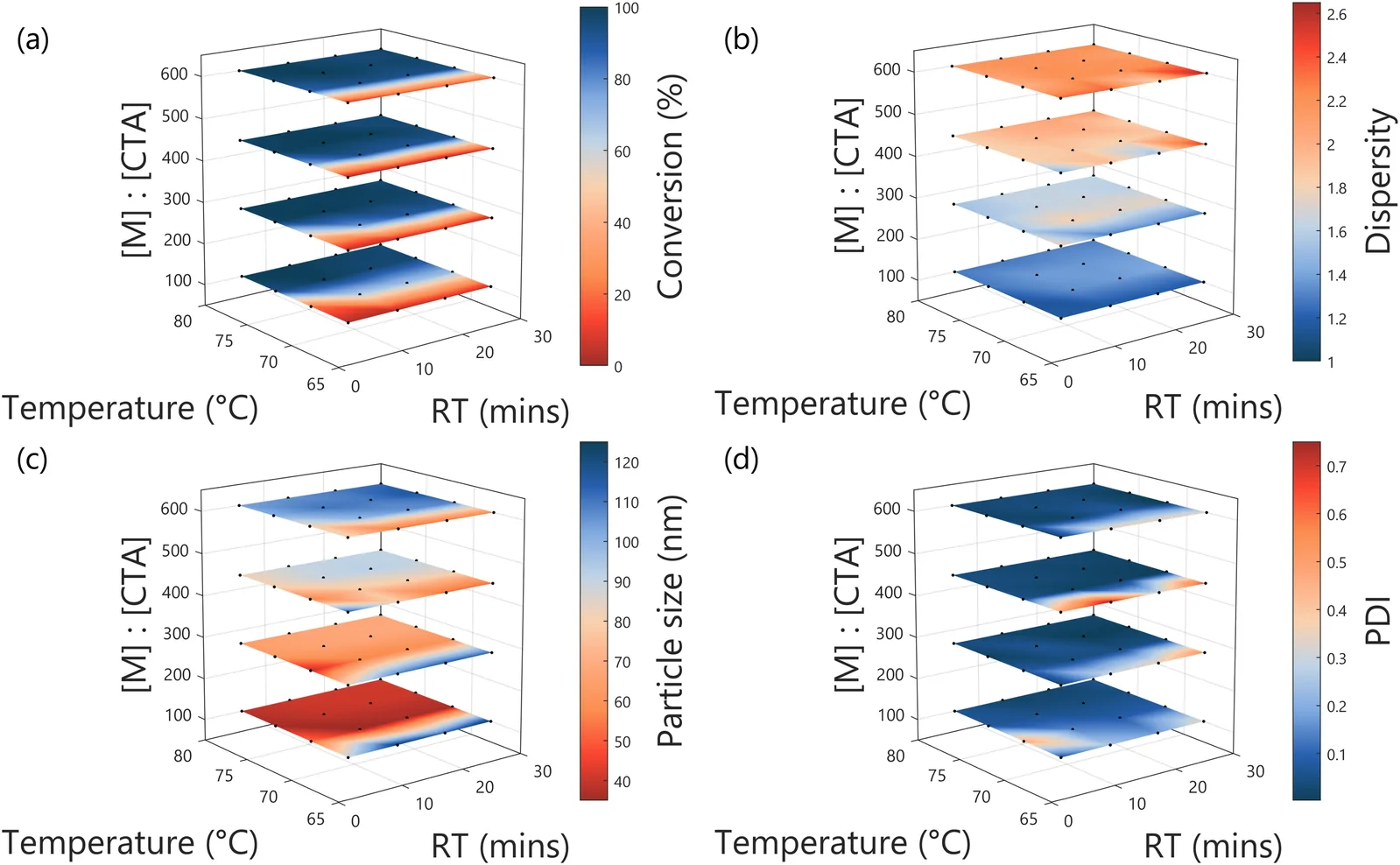
Results from the three-input full factorial DoE screen (temperature, residence time (RT) and monomer to CTA ratio ([M]:[CTA])), comprising 64 experiments, for (a) conversion, (b) molar mass dispersity, (c) particle size (nm) and (d) particle size PDI.

A programmed screen of the RAFT polymerisation of diacetone acrylamide in the presence of a poly(dimethylacrylamide)_75_ (PDMAm_75_) macro-chain transfer agent (CTA) was performed, yielding spherical particles *via* PISA ranging in size from 34 to 116 nm (Fig. 3). The temperature, residence time and ratio of monomer to macro-chain transfer agent ratio ([M] : [CTA]) were changed in a stepwise fashion to yield a 4 × 4 × 4 full factorial screen.

In this screen, all 67 (64 + 3 repeat centre points) reactions and analyses were completed in 4 days with no user interaction besides the loading of reagents, and initial selection of experimental structure following completion of each reaction, the series of analyses are each triggered once the tubular reactor reaches steady state (full analysis and details in Fig. S5–8 and Table S2 – see ESI†).

The programmed screen provides a range of benefits for this work. Firstly, it facilitated a test of reproducibility, where three repeats of the centre-point of the explored input space were performed. As is to be expected from flow chemistry, this is demonstrated to be excellent, with variability across all measured values to be extremely low, especially for monomer conversion, molar mass dispersity (*Đ*) and particle size, with the standard deviation being 2%, 2% and 1% of the found values respectively (see Table S2 and Fig. S8 in the ESI†). The greatest variability observed was for PDI, but this is due to the very low value for PDI obtained for the given conditions (17.5 min, 74 °C, target DP = 350), at an average of 0.035. At such low values, any small error (in terms of magnitude, here, 0.030) will represent a significant relative error – in this case, 85%.

Secondly, it provides macro-level understanding of the system probed, where the generalised responses for the outputs in terms of the decision variables (*i.e.* the conditions changed) can be observed. Briefly, conversion is shown to be primarily reliant upon temperature and to a lesser extent residence time. *Đ* is shown to primarily increase with higher [M] : [CTA], representing the targeting of longer polymer chains. It is worth noting that the GPC setup used a rapid column, and the polymerisation performed “through” oxygen, both combining to give a higher measured *Đ* than might be expected for a typical RAFT system, though the trend is as expected. In any case, the particles formed are well-defined throughout – as is clear from the PDI which is low wherever conversion is greater than 50%. Finally, as is to be expected, particle size is shown to be primarily dependent on the target degree of polymerisation (quantified in [M] : [CTA]) for the DAAm block, with larger particles made where longer hydrophobic polymer chains were targeted.

Finally, the rich dataset created as part of this enabled the construction of a response surface upon which a series of *in-silico* optimisation experiments could be performed, as has been demonstrated elsewhere.^35–37^ The response surface was fitted using modified Akima interpolation as part of the MATLAB fitting toolbox (for more details see ESI†). The primary purpose of this stage of the work was to evaluate the general performance of the algorithms and to act as a guide for future in-lab experiments. The relative economy of performing simulated experiments for a more statistically significant comparison of the approaches applied to the system is the critical feature.

### *In-silico* self-optimisation

The differences between the algorithms investigated here (Thompson-sampling efficient multi-objective optimisation (TSEMO),^11^ radial basis function neural network/reference vector evolutionary algorithm (RBFNN/RVEA) and a hybridised evolutionary algorithm/multi objective particle swarm optimisation (EA-MOPSO)), are outlined in Fig. 5. The key differences lie in steps 2 and 3, with the model and optimisation algorithms used. The algorithms selected use models which can handle the relatively small datasets and uncertainty associated with chemical process optimisation, based on either Gaussian process (GP)^38^ or RBFNN.^39^ A range of multi-objective optimisation algorithms were investigated to give diversity of behaviour, from the bio-inspired, heuristic particle swarm optimisation,^33^ to more conventional approaches with RVEA^40^ and non-dominated sorting genetic algorithm-II (NSGA-II).^41^ This diverse set of algorithms was able to be accessed by the platform *via* a cloud-based framework – that is the algorithms could be operated remotely from a different geographical location to the experiment. This approach allows more appropriate hardware to perform the (potentially) computationally complex algorithmic processes, and in cases where there is intellectual property sensitivity in the case of either data or algorithm, facilitates optimisations that might otherwise not be possible.

For the evaluation of the algorithms in this *in-silico* testing, the approach to optimisation selected was a direct simulation of the proposed in-lab approach in terms of methodology. The objectives for this optimisation were to maximise conversion, to minimise dispersity and PDI and to target a particle size of 80 nm. As such, this problem can be classified as a many-objective optimisation problem (MaOP). A single optimisation campaign consisted of an initial screening of 15 points using Latin Hypercube sampling (LHS) within the input space for which the responses were obtained (here, from the response surface based on the experimental data). This dataset was then provided to the selected algorithm which in turn generated a new set of inputs for the next experiment. This closed-loop methodology then proceeded iteratively until a selected endpoint was reached, after a total of 30 experiments were performed. 20 optimisation campaigns were conducted in this manner, for each of the three algorithms. A limitation of our implementation of TSEMO was that it would only accept as many objectives as there were input variables. Therefore, PDI was omitted as an objective for the running of the optimisations, since the response surface from the screen showed this was a featureless surface, as generally the particles formed were monomodal and well-defined. The exception to this was at low conversions, but this would be punished by the algorithm aiming to maximise the conversion. However, PDI was maintained as an objective in any evaluation metrics of the optimisation to allow direct comparison of plausible approaches.

**Fig. 4:**
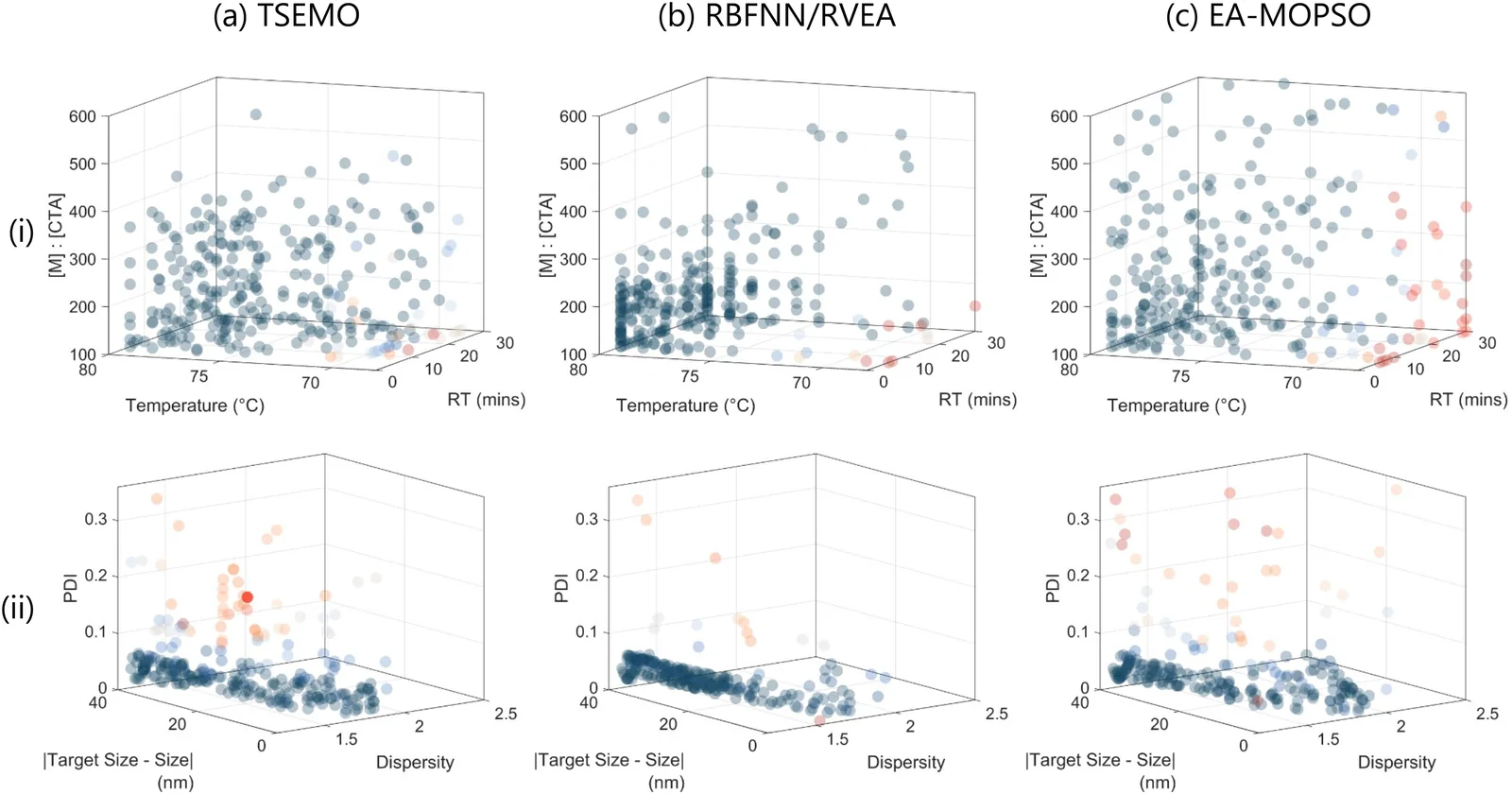
*In-silico* multi-objective optimisation for the RAFT dispersion polymerisation of DAAm in pH 2.5 water, using PDMAm_74_ as the macro-chain transfer agent and VA-044 as the initiator, in terms of (i) decision space (inputs explored) and (ii) objective space (generated data) using (a) TSEMO, (b) RBFNN/RVEA and (c) EA-MOPSO as the optimisation algorithm. The objectives were a target particle size of 80 nm, to maximise conversion and to minimise *Đ* and PDI. 20 optimisation campaigns were performed, each consisting of 15 initial LHS screening experiments (omitted for clarity) and 15 iterative algorithmically selected experiments. Each transparent datapoint represents a single selection across 300 experiments (duplicates are possible through repeated selection in each campaign).

The data from these optimisation runs qualitatively shows variations between the three algorithms in both the decision space (Fig. 4(i)) and, in turn, the resultant mapped Pareto front (Fig. 4(ii)). In particular, RBFNN/RVEA (Fig. 4b) clearly places emphasis on exploiting higher temperature experiments for lower [M] : [CTA], yielding a more detailed Pareto front where molar mass dispersity and PDI are low and conversion high, at the expense of the desired particle size (between 30 and 40 nm from the optimum).

**Fig. 5:**
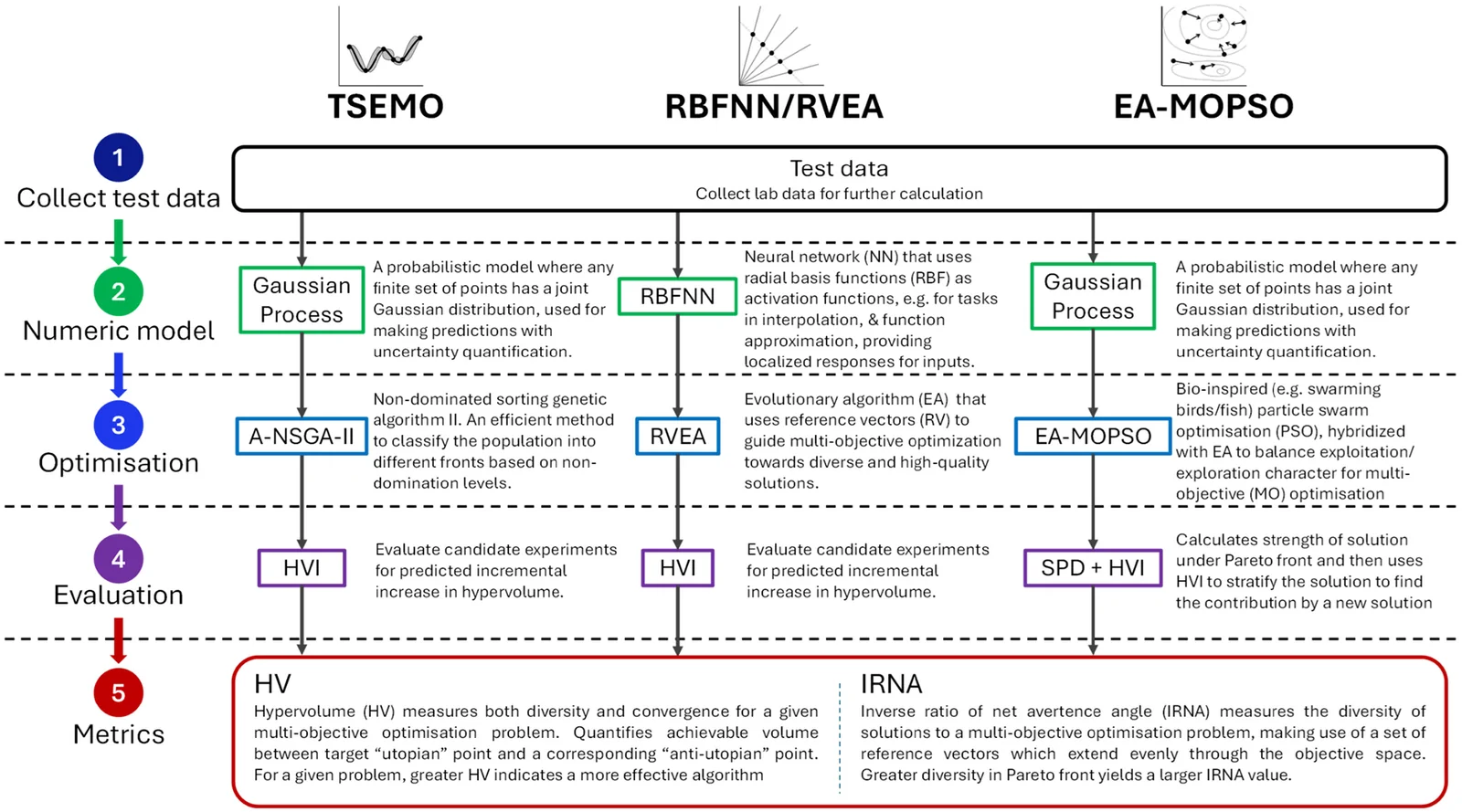
Summary of the optimisation strategies employed: Thompson sampling efficient multi-objective optimisation (TSEMO),^11^ radial basis function neural network/reference vector evolutionary algorithm (RBFNN/RVEA) and multi objective particle swarm optimisation, hybridised with an evolutionary algorithm (EA-MOPSO).^33^

TSEMO is more generalised (*i.e.* balancing exploitation with exploration) in its approach, which is illustrated by greater diversity in the decision space explored (Fig. 4a). There remains discrimination of experimental selection (*e.g.* [M] : [CTA] > 500 is almost completely dismissed by the algorithm), but a more even distribution of inputs results in a more evenly explored decision space and thus Pareto front. The EA-MOPSO results (Fig. 4c) show greater exploration in terms of the [M] : [CTA] input but is more exploitative in terms of temperature. Performance metrics are employed to assess the effectiveness of MaOP algorithms and assist decision-makers in evaluating the efficiency of optimization algorithms.^42^ Despite performance indicators potentially leading to information loss by condensing data to evaluate candidate solutions, their primary objective is to reliably and accurately capture essential information, which is crucial for MaOP problems as the number of objectives increases.

One such measure is the hypervolume, which is an essential metric in multi-objective optimisation that assesses the performance of a set of solutions by quantifying the volume of the objective space that is dominated by these solutions, relative to a reference point.^34,43^ More specifically, in this case, it measures the volume dominated between the utopian point (in this case, conversion = 100%, *Đ* = 1, particle size = 80 nm, PDI = 0) and an anti-utopian point at the opposite end of these scales (conversion = 0%, *Đ* = 3, PDI = 1 and where the loss function used for the size objective = 2; for more details see eqn (S1) (ESI)†). The hypervolume metric quantifies the quality and diversity of the Pareto front by producing a single scalar number. A higher hypervolume score indicates a more accurate approximation of optimal solutions. The calculation of this metric can be computationally costly, particularly in higher dimensions. However, it is crucial for comparing various optimisation strategies and promoting a wide range of solutions.

**Fig. 6:**
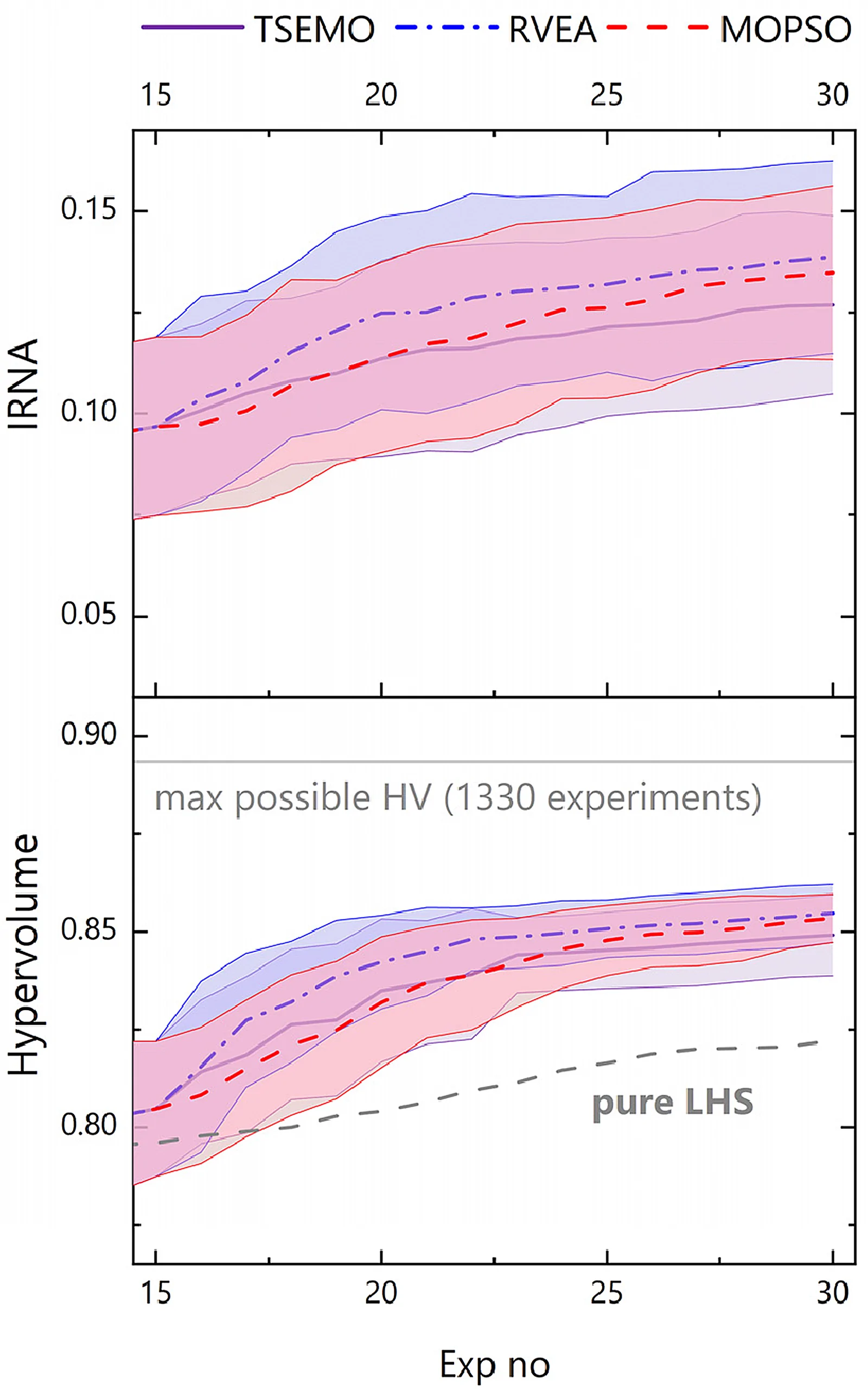
Mean evolution of hypervolume and inverse ratio of net avertence angle (IRNA) for the 20 optimisation campaigns for TSEMO, RBFNN/RVEA and EA-MOPSO. For hypervolume, the mean for 20 campaigns of 30 experiment Latin Hypercube sampling (LHS) of the decision space is also shown. The shaded area represents one standard deviation.

The average evolution of hypervolume across the 20 runs for each algorithm (Fig. 6) shows similar performance across each of the algorithms, *i.e.* all (as expected) improve on a 30 experiment Latin Hypercube sampling (LHS) of the decision space. TSEMO was found to slightly underperform in terms of hypervolume, but the performance is shown to be similar when accounting for uncertainty, with overlapping uncertainties (which show standard deviation). Furthermore, caution must be applied in interpretation here, not least because of the four-dimensional nature of the data, meaning hypervolume is not trivially visualised. In addition, the measurement does not tell the whole story, as in higher dimensions the discriminative power of HV is significantly reduced.^44^

Another aspect in measuring the effectiveness of each algorithm is the diversity of the Pareto front generated. Inverse ratio of net avertence angle (IRNA) is a metric of purely this diversity.^44^ It is important to use more than one metric to measure the success of optimisations when considering four or more objectives, as hypervolume may be misleading in these cases.^44^ Here, IRNA gives a similar trend to that seen for hypervolume, supporting the conclusions already drawn. It is worth noting the high level of error across the range of experiments for IRNA, which is intrinsic to the relatively data-poor nature of a 30-experiment optimisation, where a relatively small number of experiments may make up the Pareto front.

In light of the above, it is important to consider the visualised data and available metrics (HV, IRNA) in concert to provide a well-rounded characterisation of each optimisation campaign.

### In-lab self-optimisation

**Fig. 7:**
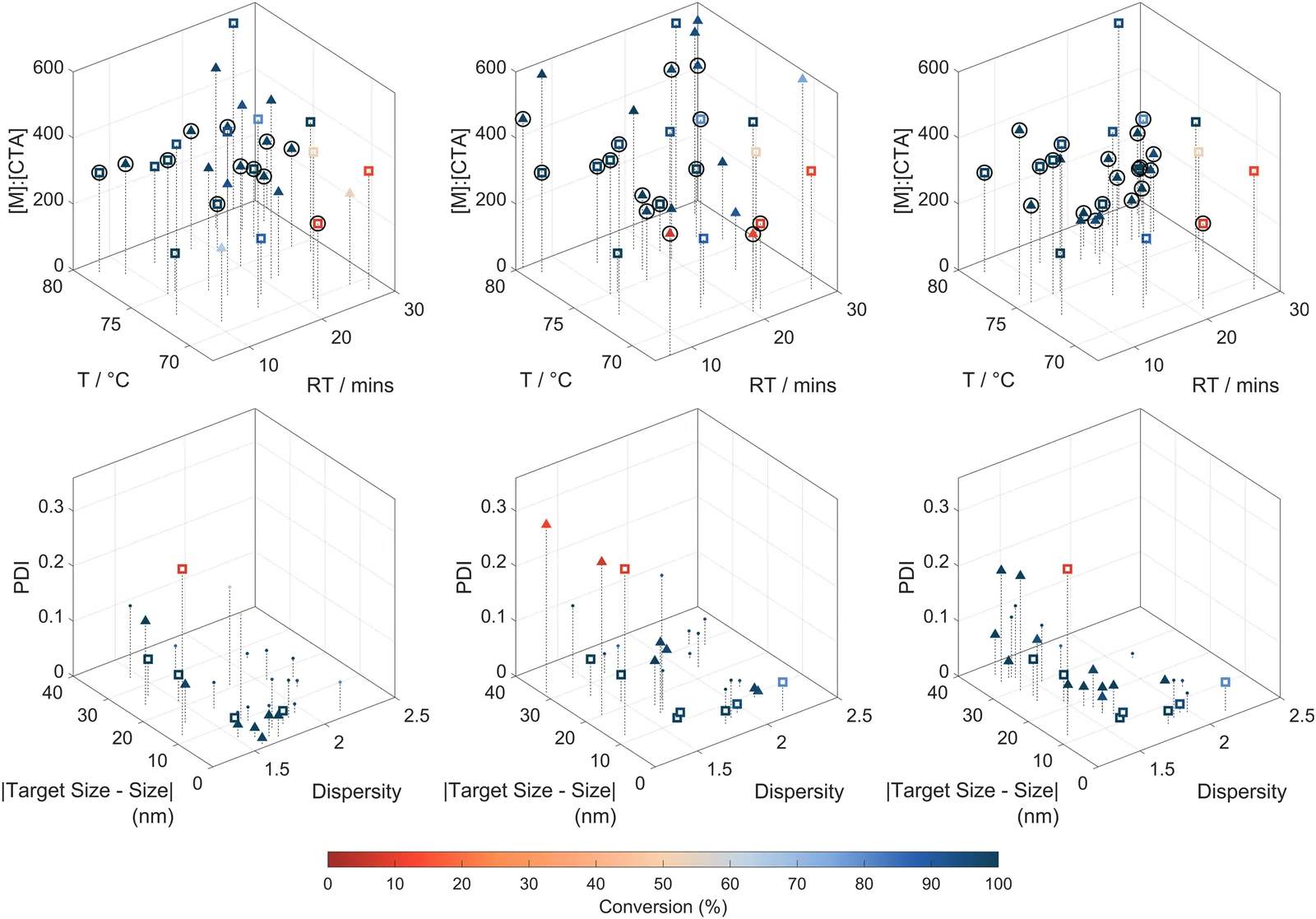
Comparison of the three optimisation algorithms selected for this work in targeting 80 nm particles (a) TSEMO, (b) RBFNN/RVEA and (c) EA-MOPSO, while maximising conversion and minimising dispersity and PDI. The initial 15 experiments are identical in each of the cases, generated by Latin Hypercube Sampling (shown as empty squares), with the algorithmically selected experiments shown as filled triangles. Row (i) shows the experiments in terms of decision space (*i.e.* conditions used), with those of the Pareto set circled and row (ii) shows the data in terms of objective space (*i.e.* measured properties). In row (ii), dominated solutions are shown only as small dots, with only those points on the Pareto front shown as squares/triangles.

In any case, the data in its entirety shows promise for each of the algorithms which outperform the pure LHS and exhibit qualitatively observable diversity of behaviour, making all three suitable candidates to be taken forward for application in-lab. A new batch of PDMAm macro-CTA was synthesised, and successful exploration of the Pareto front using closed-loop optimisation demonstrated across the three examples (Fig. 7). It is worth noting that since a new starting material was used, the results are expected to be comparable to those from the screen, but not identical.

There were again notable variations in algorithmic behaviour across the three optimisations. For example, the distribution of the experiments with regards to input space is shown to be more focussed on a narrower search space for the EA-MOPSO optimisation, with increasing diversity for TSEMO and even more so for the RBFNN/RVEA. The results in terms of objective space qualitatively reflect this input diversity – where the more clustered search by the EA-MOPSO algorithm yields a larger number of non-dominated solutions in a smaller space.

**Fig. 8:**
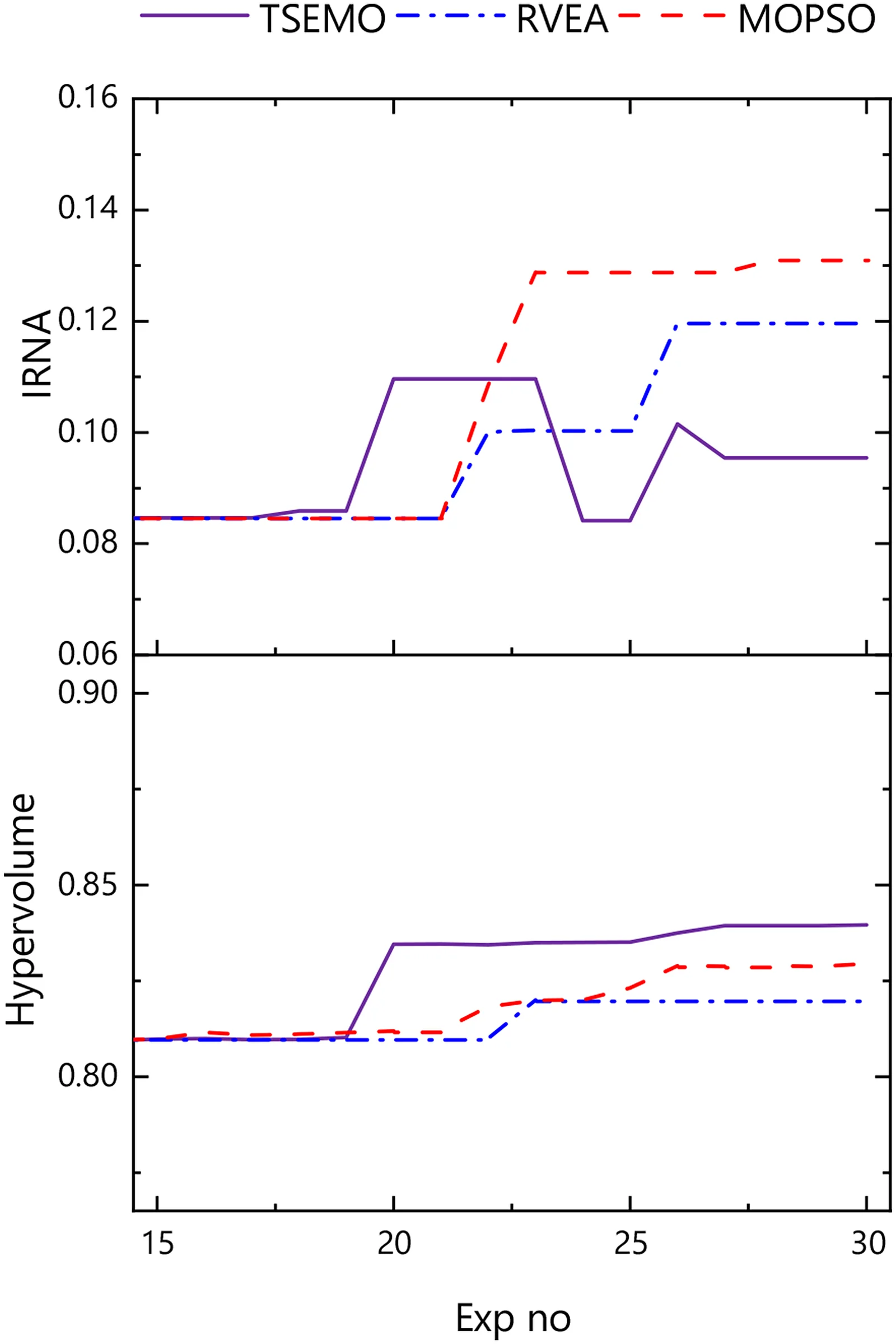
Hypervolume and IRNA evolution for the three in-lab optimisation approaches selected for this work in targeting 80 nm particles (a) TSEMO(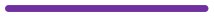), (b) RBFNN/RVEA(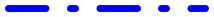) and (c) EA-MOPSO(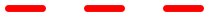), while maximising conversion and minimising dispersity and PDI.

The metrics from the in-lab testing show a different trend in hypervolume compared to the initial *in-silico* testing (Fig. 8). TSEMO in this case outperforms both the other two algorithms, largely due to a single experiment at iteration 20 (28 min, 78 °C, [M] : [CTA] = 135). RBFNN/RVEA particularly gave a lesser improvement in hypervolume than might be expected given the prior mean *in-silico* data, but at least a partial justification is found in the magnitude of the uncertainty in those *in-silico* plots. The number of runs permissible from a cost perspective in-lab is clearly much lower than that with an emulated approach; and so, the possibility of finding one of the less successful pathways for a single run remains. Furthermore, from post-experiment analysis, this can be attributed to poor performance in modelling on the real-life data, and as such, in-lab, the algorithm selected experiments from across the reaction space rather than giving the same exploitative performance observed *in-silico*.

Another important consideration is to weigh the merit of solely evaluating the success of a given optimisation in terms of hypervolume. In terms of the objectives of the experiment, each of the optimisation campaigns here are successful in giving a skilled user much of the necessary information to select conditions which provide them with a polymer particle with a set of desired properties. There are subtle differences, and to an extent we see an algorithm with more emphasis placed upon exploitation in EA-MOPSO compared to algorithms which appear to show more exploration in TSEMO and in the in-lab example, RBFNN/RVEA – though for the landscape provided by the *in-silico* model, this is not demonstrated for RBFNN/RVEA. This then enables the user to select an algorithm based on their needs. For example, the balance between exploration and exploitation achieved by TSEMO may make this algorithm more suited to manufacturing applications, since its exploration gives a greater idea of the size of regions of stable output. Where exploitation is of more interest to the user than the balanced approach described, the application of EA-MOPSO over TSEMO in-lab may be of more interest. Despite struggling in-lab, the successful exploitative performance of RBFNN/RVEA on the smoother, continuous *in-silico* surfaces suggests that this algorithm is suitable for optimisation on models generated by full-factorial screens, or indeed other such datasets – such as those from computational flow dynamics (CFD) simulations.

Given this diversity of algorithmic behaviour, we would emphasise the opportunity that the platform technology provides in terms of the diversity of possible approaches and the understanding that the different approaches might offer. For example, a 67-experiment screen would in many cases be a too time-consuming affair; but given that the user time to produce the data here was less than a single day's work, the comprehensive nature of the data would well be attractive in cases where the feedstock chemicals were relatively affordable. Conversely, were the raw materials more expensive, an algorithmic approach may be more desirable, with much of the information available after a campaign of fewer than half the number of experiments. This is not to say these two extremes are the only viable approaches – a less detailed screen, or a hybridised approach, using a screen as the basis for further self-optimisation offer additional plausible strategies.

## Conclusions

In this work we have demonstrated a range of approaches to explore and optimise the complexities of a particle-forming polymerisation system. The platform gave unprecedented diversity of information for automated polymer synthesis, facilitating days-long unsupervised experiments with accompanying ^1^H NMR spectroscopy, GPC and DLS data. A screen of 67 experiments gave a rich dataset in just four working days. From the resultant dataset, *in-silico* optimisation studies were performed, confirming the validity of AI-guided optimisation. Machine learning algorithms (TSEMO, RBFNN/RVEA and EA-MOPSO) were accessed *via* a cloud-based framework, and used to target a particle size, while maximising conversion and minimising both molar mass dispersity and particle PDI, both *in-silico* and in-lab – providing elucidation of the Pareto front. A range of algorithmic behaviour was observed, and example applications for each algorithm identified. As such, a significant step towards particles-on-demand is taken, which could find application across polymerisation techniques.

This work highlights key challenges faced by the chemists engaging with automation, AI-guided optimisation, and further, the complications that are introduced for many-objective problems. It is imperative that we engage with effective characterisation of the optimisation process, using appropriate performance indicators in conjunction with clear visualisation of the experimental data. Furthermore, we must be careful to consider the range of approaches which are made possible by autonomous platforms, comparing the relative merits of automated screens, AI-guided optimisation, and hybridised methodologies.

## Author contributions

STK: data curation, investigation, methodology, software, visualization, writing – original draft, writing – review & editing, conceptualization, funding acquisition; KEW: data curation, investigation, methodology, software, visualization, formal analysis, writing – review & editing; NI: investigation, methodology, software, writing – review & editing; RAOC: investigation, visualization, writing – review & editing; PP: investigation, writing – review & editing, KC: investigation, software, writing – review & editing; JO: conceptualization, supervision, funding acquisition, writing – review & editing; GP: conceptualization, supervision, funding acquisition, writing – review & editing; TWC: conceptualization, supervision, funding acquisition, writing – review & editing; RAB: conceptualization, software, supervision, funding acquisition, writing – review & editing; NJW: conceptualization, funding acquisition, project administration, supervision, writing – review & editing.

## Conflicts of interest

There are no conflicts of interest to declare.

## Supplementary Material

PY-016-D5PY00123D-s001

## Data Availability

The data supporting this article have been included as part of the ESI.†
