# Supplementary material for: Self-driving laboratory platform for many-objective self-optimisation of polymer nanoparticle synthesis with cloud-integrated machine learning and orthogonal online analytics
Source: Polym Chem. 2025 Feb 11;16(12):1355–64. doi: 10.1039/d5py00123d (PMC13580201; doi:10.1039/d5py00123d)
Supplement: Supplementary file 1 [file PY-016-D5PY00123D-s001.pdf]

Supplementary Information (SI) for Polymer Chemistry.
This journal is © The Royal Society of Chemistry 2025

Supporting information (SI) for:

Self-driving laboratory platform for many-objective self-optimisation of polymer nanoparticle synthesis with cloud-integrated machine learning and orthogonal online analytics.

*Stephen T. Knox, Kai E. Wu, Nazrul Islam, Roisin O'Connell, Peter Pittaway, Kudakwashe Chingono, John Oyekan, George Panoutsos, Thomas W. Chamberlain, Richard A. Bourne, Nicholas J. Warren**

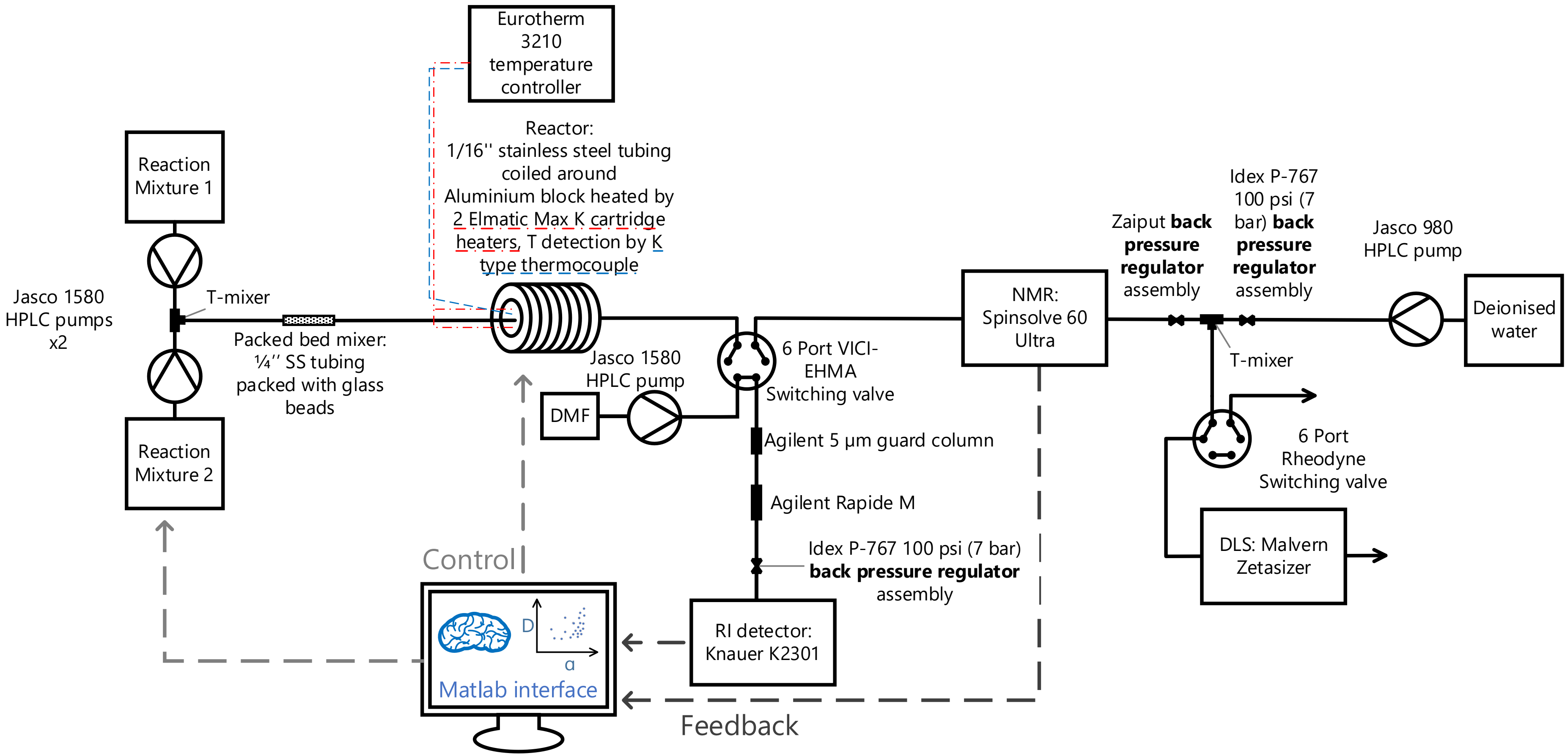

Figure S1. Schematic for the platform used in this work.

Platform

The MATLAB-controlled automated platform consisted of two Jasco PU-1580 HPLC pumps; a PFA tubular reactor (0.7 mm, I.D, 2 mL), coiled around an aluminium block heated by a Eurotherm 3210 controller fitted with two Elmatic Max K cartridges, with a Zaiput backpressure regulator (pressurised to 4 bar); a Spinsolve benchtop NMR instrument, a custom-built GPC instrument, as described in our previous work;[14] and a Malvern Zetasizer DLS instrument (Figure S1).

Online ^{1}H NMR spectroscopy

NMR spectra were recorded using a Magritek Spinsolve 60 Ultra. They were collected using a presaturation solvent suppression routine (1s saturation pulse at 4.79 ppm of -68 dB, 7 μs excitation pulse, spectral width of 5 kHz (32,768 points), acquisition time of 6.4 s, repetition time of 10 s and number of scans = 2) All chemical shifts are reported in ppm (δ).

Online gel permeation chromatography (GPC)

The custom-built GPC setup was constructed using a Jasco PU-1580 HPLC pump (flow rate: 2 ml min^{-1}), GPC columns (Agilent Rapide M plus guard) and a Knauer 2301 refractive index (RI) detector, all controlled by a homemade MatLab program. DMF eluent was used containing LiBr (1 % w/v). The program records the time of injection from the triggering of the switching valve, and the subsequent RI trace. Molecular weights can then be calculated from calibration to a series of near-monodisperse standards (PMMA – M_p: 885–2,200,000 g mol^{-1}). The injection volume is approximately 3 μl.

To improve the accuracy of the GPC data acquired, a triple injection sequence was introduced (injected at 175 s intervals). This yielded three peaks to analyse and average analytical information from. Each peak is analysed according to its injection time.

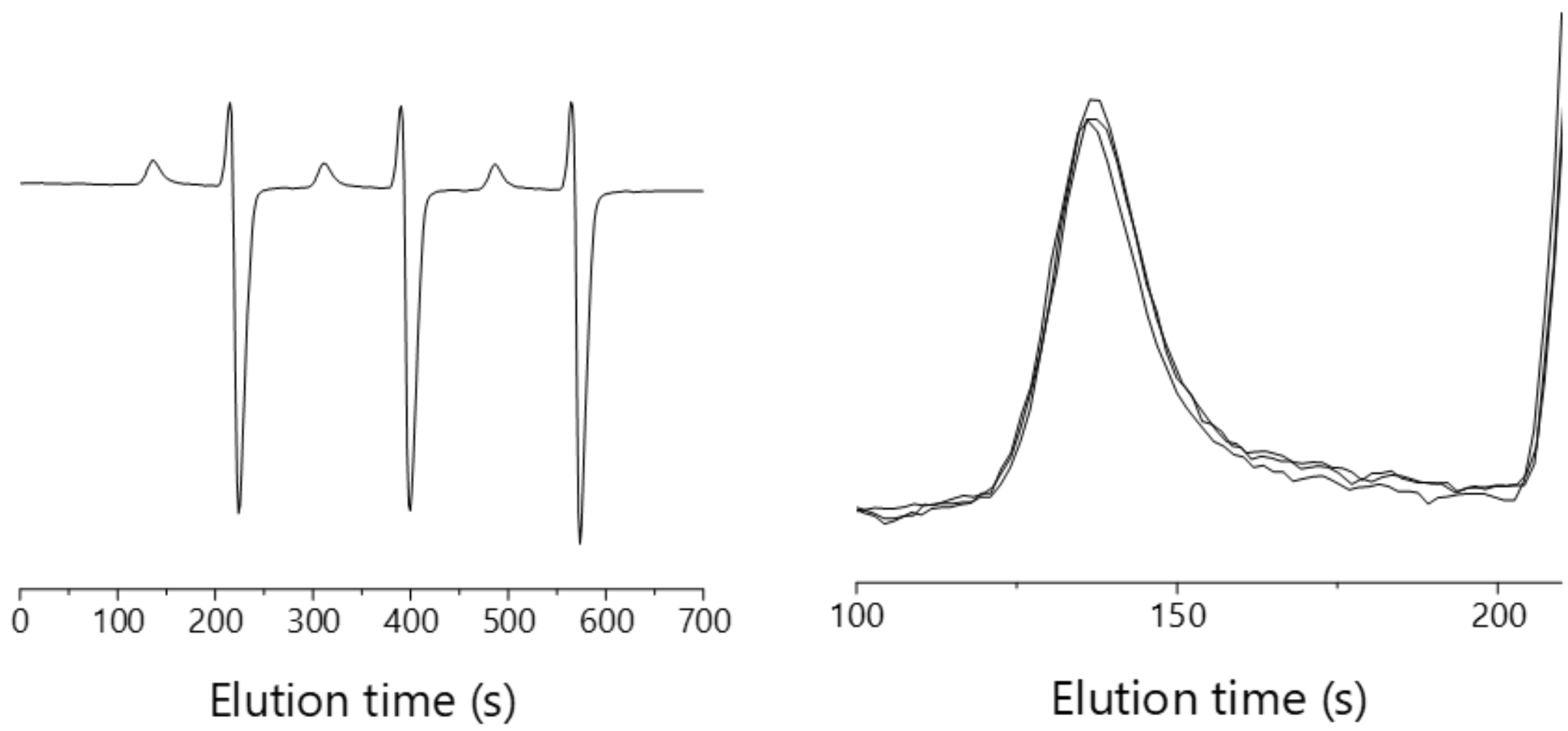

(a) (b)

Figure S2. (a) Full raw chromatogram obtained from triple injection sequence from gel permeation chromatography. (b) Resultant chromatograms as calibrated to injection time.

Online Dynamic Light Scattering

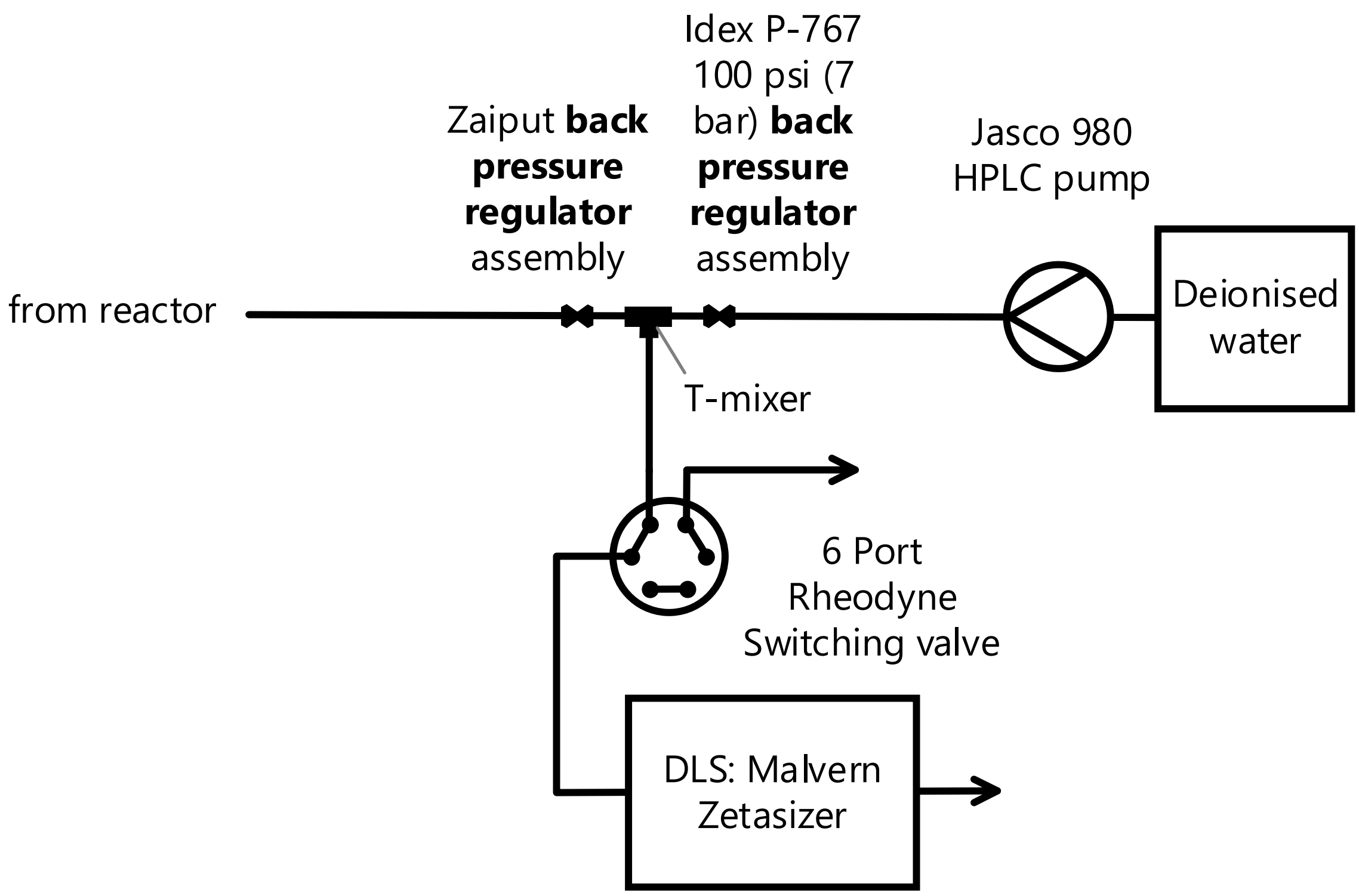

Figure S3. Schematic for the online dynamic light scattering analysis performed in this work

To incorporate DLS into the automated platform, a flow cell (ZEN0023 quartz cell, Hellma) was inserted into the Malvern instrument and attached to the flow stream, after a switching valve, in addition to a dilution stream to bring the product stream to the appropriate concentration for analysis. While the platform was brought to steady state, the flow was set to bypass the instrument. Upon triggering of the measurement, the dilution pump set to 5 ml min^{-1}, the total flow rate of the product stream to 0.1 ml min^{-1} and the flow redirected to pass through the flow cell, for 30 seconds, bringing the mixture to steady state.

In the absence of the ability to trigger the instrument remotely from an external program, the instrument recorded a measurement every 3 minutes and the most recent measurement acquired by the GUI. The measurement itself relied upon 5 runs of 10 seconds each, after 30s of temperature equilibration followed by the instrument's autonomous measurement optimisation. After waiting for over 2 minutes from the point of the flow being stopped, the most recent measurement was accessed by the GUI and the key information extracted for continued use.

Mass balances and flow rate calculations

For the calculation of mass balances, we begin with the desired properties of the final product. Of that product we know the Target DP (DP), the residence time (τ), the weight percent (w), and the concentration of initiator ($[ini]$), which is constant throughout. In all mass balances, a density of 1 g cm^{-1} is assumed since all reagents are in a low concentration in water.

We can construct a simple model for the mixing of two reagent streams with flowrate, Q and concentrations, x as follows:

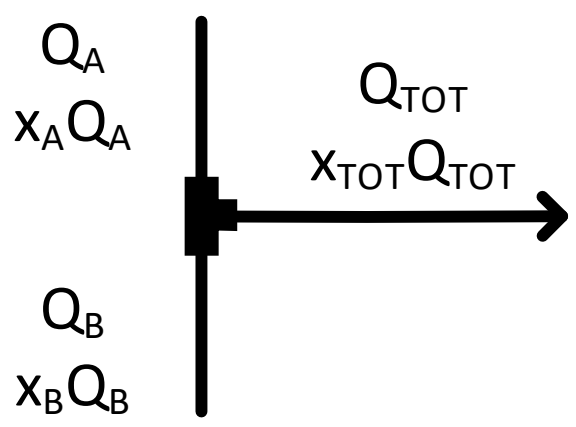

$$Q_{TOT} = \frac{V_R}{\tau}$$

$$Q_A + Q_B = Q_{TOT}$$

$$x_AQ_A + x_BQ_B = x_{TOT}Q_{TOT}$$

$$x_AQ_A + x_B(Q_{TOT} - Q_A) = x_{TOT}Q_{TOT}$$

$$x_AQ_A + x_BQ_{TOT} - x_BQ_A = x_{TOT}Q_{TOT}$$

$$Q_A(x_A - x_B) + x_BQ_{TOT} = x_{TOT}Q_{TOT}$$

$$Q_A(x_A - x_B) = x_cQ_{TOT} - x_BQ_{TOT}$$

$$Q_A(x_A - x_B) = Q_{TOT}(x_{TOT} - x_B)$$

$$Q_A = \frac{Q_{TOT}(x_{TOT} - x_B)}{(x_A - x_B)}$$

Since all of the concentration values initially calculated as mass concentrations are scaled by the same molecular weight, then we can calculate the flow rate of pump A (Q_A) as

$$Q_A = \frac{Q_{TOT}([CTA] - [CTA]_B)}{[CTA]_A - [CTA]_B} \quad (1)$$

And the flow rate of pump B (Q_B) as

$$Q_B = Q_{TOT} - Q_A \quad (2)$$

Therefore, to calculate the desired flowrates, we then need to calculate the final desired concentration of CTA ($[CTA]$) in terms of the desired DP and the known chemical compositions of the two feedstock solutions.

Starting with the target DP we can calculate the mass of CTA required,

$$DP = \frac{n_{mon}}{n_{CTA}} = \frac{m_{mon} \times \frac{1}{MW_{mon}}}{m_{CTA} \times \frac{1}{MW_{CTA}}}$$

$$\frac{m_{mon}}{m_{CTA}} = \frac{DP \times MW_{mon}}{MW_{CTA}}$$

$$m_{mon} = \frac{DP \times MW_{mon}}{MW_{CTA}} \times m_{CTA}$$

$$m_s = m_{ini} + m_{CTA} + m_{mon}$$

$$m_s - m_{ini} = m_{CTA} + m_{mon} = m_{CTA} + \frac{DP \times MW_{mon}}{MW_{CTA}} \times m_{CTA} = m_{CTA}\left(1 + \frac{DP \times MW_{mon}}{MW_{CTA}}\right)$$

And therefore

$$m_{CTA} = \frac{m_s - m_{ini}}{\left(1 + \frac{DP \times MW_{mon}}{MW_{CTA}}\right)}$$

If we arbitrarily assign m_{TOT} as 1;

$$w = \frac{m_s}{m_{TOT}} = m_s$$

This gives

$$m_{CTA} = \frac{w - m_{ini}}{\left(1 + \frac{DP \times MW_{mon}}{MW_{CTA}}\right)}$$

The mass of initiator can be calculated simply from the desired concentration of initiator ([ini])for the experiment – in this case, 0.75 mmol dm^{-3}. For water, the density (ρ) can be approximated to one and again taking the arbitrary value for m_{tot} as one, we can substitute:

$$m_{ini} = [ini] \times m_{TOT} \times \rho \times MW_{ini} = [ini] \times MW_{ini}$$

$$m_{CTA} = \frac{w - ([ini] \times MW_{ini})}{\left(1 + \frac{DP \times MW_{mon}}{MW_{CTA}}\right)}$$

$$[CTA] = \frac{w - ([ini] \times MW_{ini})}{\left(1 + \frac{DP \times MW_{mon}}{MW_{CTA}}\right)} \times \frac{1}{MW_{CTA}} = \frac{w - ([ini] \times MW_{ini})}{(DP \times MW_{mon} + MW_{CTA})} \qquad (3)$$

The combination of above can be used to conveniently calculate the pair of flow rates required for a given residence time (τ), target DP (DP) and the known chemical composition of the two reagent feedstock solutions, described with subscripts A and B. All concentrations are denoted as "$[x]$" and are used in units of $mol\ dm^{-3}$.

$$Q_A = \frac{\frac{V_R}{\tau}\left(\frac{w - ([ini] \times MW_{ini})}{(DP \times MW_{mon} + MW_{CTA})} - [CTA]_B\right)}{[CTA]_A - [CTA]_B} \quad (4)$$

$$Q_B = Q_{TOT} - Q_A \quad (2)$$

These equations were integrated into the MATLAB GUI used to control all the experiments performed using the platform.

It is also important to know the final monomer concentration ($[M]_f$), so that an accurate conversion can be calculated from the NMR spectrum generated. This can be found simply by use of

$$x_A Q_A + x_B Q_B = x_{TOT} Q_{TOT}$$

Where if we consider x in this case to represent the concentration of monomer, then the total concentration of monomer is given by

$$\frac{x_A Q_A + x_B Q_B}{Q_{TOT}} = x_{TOT} \quad (5)$$

Conversion methodology

Benchtop ^{1}H NMR spectroscopy uses a fixed receiver gain and therefore the integral of signal is directly proportional to the concentration of protons in a given volume. In this case, the same region of flow cell is maintained throughout, meaning volume is constant. This enables the calculation of conversion from a single region, with no requirement for normalisation of signal. A calibration curve was produced to elucidate the relationship between monomer concentration and the integral for the alkenyl protons at 6.6-5.3 ppm. This enables forward prediction of the integral that would be expected for a given target DP at t = 0, which can be inserted into Equation (6) to yield conversion.

The platform was operated in the same fashion as for the main experiments, with two mixed streams: two "dummy" reactant solutions were formulated without initiator and loaded onto the platform. Using Equations (2) & (4), the appropriate pair of flow rates were calculated for a range of 10 monomer concentrations between $2.85 \times 10^{-4}\, mol\, dm^{-3}$ and $4.2 \times 10^{-4}\, mol\, dm^{-3}$. These conditions loaded to the pumps and the system brought to steady state, before 3 NMR spectra were collected and the integral between 5.3 and 6.6 measured (black squares, Figure S4). Prior to the screening experiment, individual t_0 experiments for the four target DPs were performed, and these can be used as a validation for this approach, and indeed they show the predictive power of the calibration curve (blue crossed circles, Figure S4), by each falling on or very near to the line.

Table S1. Flow rates to achieve desired monomer concentrations from 2.85 × 10^{-4} mol dm^{-3} to 4.2 × 10^{-4} mol dm^{-3}

**Target [M] / mol dm^{-3}**	**Q_A / ml min^{-1}**	**Q_B / ml min^{-1}**
2.85×10^4	0.970	0.030
3×10^4	0.873	0.127
3.15×10^4	0.775	0.225
3.3×10^4	0.677	0.323
3.45×10^4	0.580	0.420
3.6×10^4	0.482	0.518
3.75×10^4	0.384	0.616
3.9×10^4	0.287	0.713
4.05×10^4	0.189	0.811
4.2×10^4	0.091	0.909

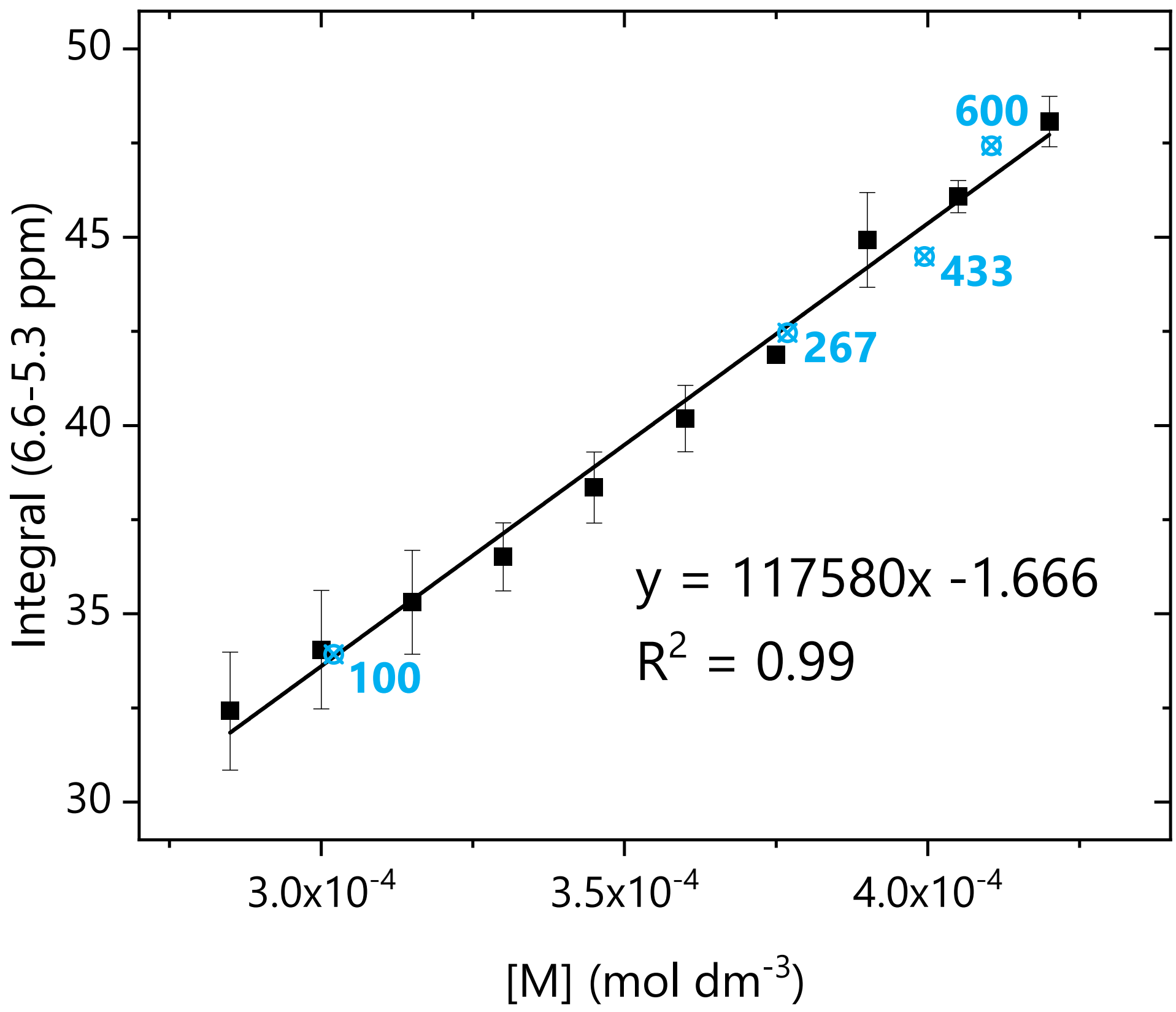

Figure S4. Calibration curve relating the alkenyl integral at 6.6-5.3 ppm to the monomer concentration ([M]). Each point is the average of three measurements, with error bars indicating the minimum and maximum values obtained for each of the three. The blue crossed circles show the integral value obtained for t_0 samples for each of the four levels of the 4 x 4 x 4 screen using the reagent solutions prior to the screen. Their proximity to the calibration curve demonstrates the applicability of this approach. Inset: The equation for the calibration curve and R^2 value.

As shown on the graph, the calibration equation is

$$y = 1.18 \times 10^6 \cdot x - 1.666$$

Where x is given as the concentration of monomer as calculated by Equation (5).

We can then insert this into the conventional conversion equation to calculate the conversion from any given integral,

$$\alpha = 1 - \frac{[M]}{[M]_0} = 1 - \frac{int_m}{1.18 \times 10^6 \cdot x - 1.666} \qquad (\mathbf{6})$$

where x is the calculated monomer concentration for the reaction mixture assuming no reaction has taken place.

Materials

2,2-azobis(2-methylpropionitrile) (AIBN, 98 %), dimethylacrylamide (DMAm, 99 %), 1,4-dioxane, diethyl ether (Sigma-Aldrich (UK)); 2,2′-Azobis[2-(2-imidazolin-2-yl)propane]dihydrochloride (VA-044, Wako Speciality Chemicals); diacetone acrylamide (DAAm, 99 %, Alfa Aesar); 2-(Butylthiocarbonothioylthio)propanoic acid (TTC-1, ≥95 %, Boron Molecular (Raleigh, USA)), were all used as supplied.

Methods

Synthesis of PDMAm$_x$ macro chain transfer agent (macro-CTA)

AIBN (21.6 mg, 0.13 mmol), TTC-1 (0.60 g, 2.51 mmol) and DMAm (24.9 g, 0.251 mol) were dissolved with stirring in 61.6 mL 1,4-dioxane for a 30 % solids (w/w) solution which was sealed and sparged with N_2 for 30 minutes. The solution was heated with stirring to 70 °C for 46 minutes, to target a conversion of 75 %, after which it was cooled to room temperature and then exposed to air to quench further reaction. The resulting polymer was precipitated from solution by dropping into vigorously stirred diethyl ether (1.4 L) and dried under reduced pressure to yield PDMAm$_{75}$ in run 1 and PDMAm$_{74}$ in run 2, as determined by ^{1}H NMR, assuming all conversion of monomer yielded polymer.

Automated RAFT polymerisation

VA-044, PDMAm$_x$ macro-CTA (macro-chain transfer agent) and DAAm were loaded into two reservoir solutions in ratios to approximately target degrees of polymerisation (DP) of 80 and 2500 respectively and dissolved in pH 2.5 water at 7.5 % solids (w/w). E.g. **Reservoir 1** (Target DP = 80) : VA-044 (20.7 mg, 0.06 mmol), PDMAm$_{75}$ macro-CTA (2.31 g, 0.30 mmol) and DAAm (4.06 g, 24.0 mmol) in 79 mL pH 2.5 water; **Reservoir 2** (Target DP = 2495): VA-044 (46.7 mg, 0.14 mmol), PDMAm$_{75}$ macro-CTA (0.261 g, 0.30 mmol) and DAAm (14.30 g, 84.5 mmol) in 180 mL pH 2.5 water. For each experiment approximately 10.5 mL in total was used (2 mL initial purge, 7 mL for steady state, 1.5 mL for analysis), with the appropriate flow rates from each reservoir calculated to target a particular DP.

Detailed experimental protocol for optimisation experiments

VA-044, PDMA$_x$ macro-CTA and DAAm were loaded into two reservoir solutions in ratios to approximately target degrees of polymerisation of 80 and 2500 respectively and dissolved in pH 2.5 water. Each delivery pump is then primed with the material. The chemical information for the formulation used was loaded to the GUI, in order that the appropriate flow rates for a target DP can be calculated, as well as the total monomer concentration, that conversion can be calculated from the calibration curve. The limits (min/max/step size) of the experiment in terms of input variables were then provided the platform and the experiment started.

For each experiment iteration within an automated experiment, the experimental protocol is as follows: for the given conditions (by full factorial sampling, initial Latin Hypercube sampling or algorithmic selection), the reactor is brought to the correct temperature, and the appropriate flow rates calculated as per Equations (2) & (4). After reaching the selected temperature, the reaction mixtures were pumped at the same ratio to that calculated for the experiment but scaled to a total flow rate of 1 ml min^{-1}, for an initialisation period of two minutes. This facilitated the equilibration of the output of the mixer (i.e. at the appropriate

ratio for the given experiment) and aided the clearing out of the reactor after the previous experiment. Following this, the flow rates were then reduced to the appropriate flow rates for the required residence time. These conditions were maintained for 3.5 times the residence time and then the analytical element of the experiment performed.

First, the GPC analysis would be started, with the triggering of the sample loop. The GPC analysis used three injections and upon the third injection, the flow was altered (again maintaining the flow ratio for the experiment) to 0.1 ml min^{-1}. The NMR and DLS analyses loops were then started. For the NMR, the spectrometer would first perform a "shim on sample" and then the NMR experiment proper – using the presaturation routine described above. For the DLS, the appropriate sample loop (SL2) was switched to direct the flow through the DLS flow cell and then the DLS dilution pump turned on at 5 ml min^{-1} for 30 seconds. The flow was then redirected, and the dilution pump switched off. The analysis was performed as described above, and upon the completion of all three analyses, the next iteration of the loop was triggered, again by selecting the experimental conditions and setting the reactor temperature.

The maximum number of iterations was set at 15 for each algorithmically driven element, with each experiment using the same initial 15 LHS starting experiments, making each "optimisation" 30 experiments in total. The data was passed to the algorithms as input / output pairs of conditions selected / results obtained, with particle size converted to a size objective *via* a loss function to target a particle size of 80 nm:

$$Size\ Objective = \left(\frac{Size - Target\ Size}{Target\ Size}\right)^2$$

Equation S1. Loss function used for size targeting as part of the optimisation campaigns

For TSEMO this was performed in MATLAB, and the objectives were passed as the natural logarithm of the values, as advised in the original use case. For RBFNN/RVEA and EA-MOPSO, input/output pairs and new inputs were passed via .txt files uploaded to a shared cloud-based directory.

Algorithmic application

TSEMO is a well-established algorithm and was used in MATLAB, as available here, https://github.com/Eric-Bradford/TS-EMO.[2,3]

The RBFNN/RVEA was applied based upon code modified from PlatEMO,[4] in ask-tell mode.

EA-MOPSO was applied as described in previous work,[5] in ask-tell mode.

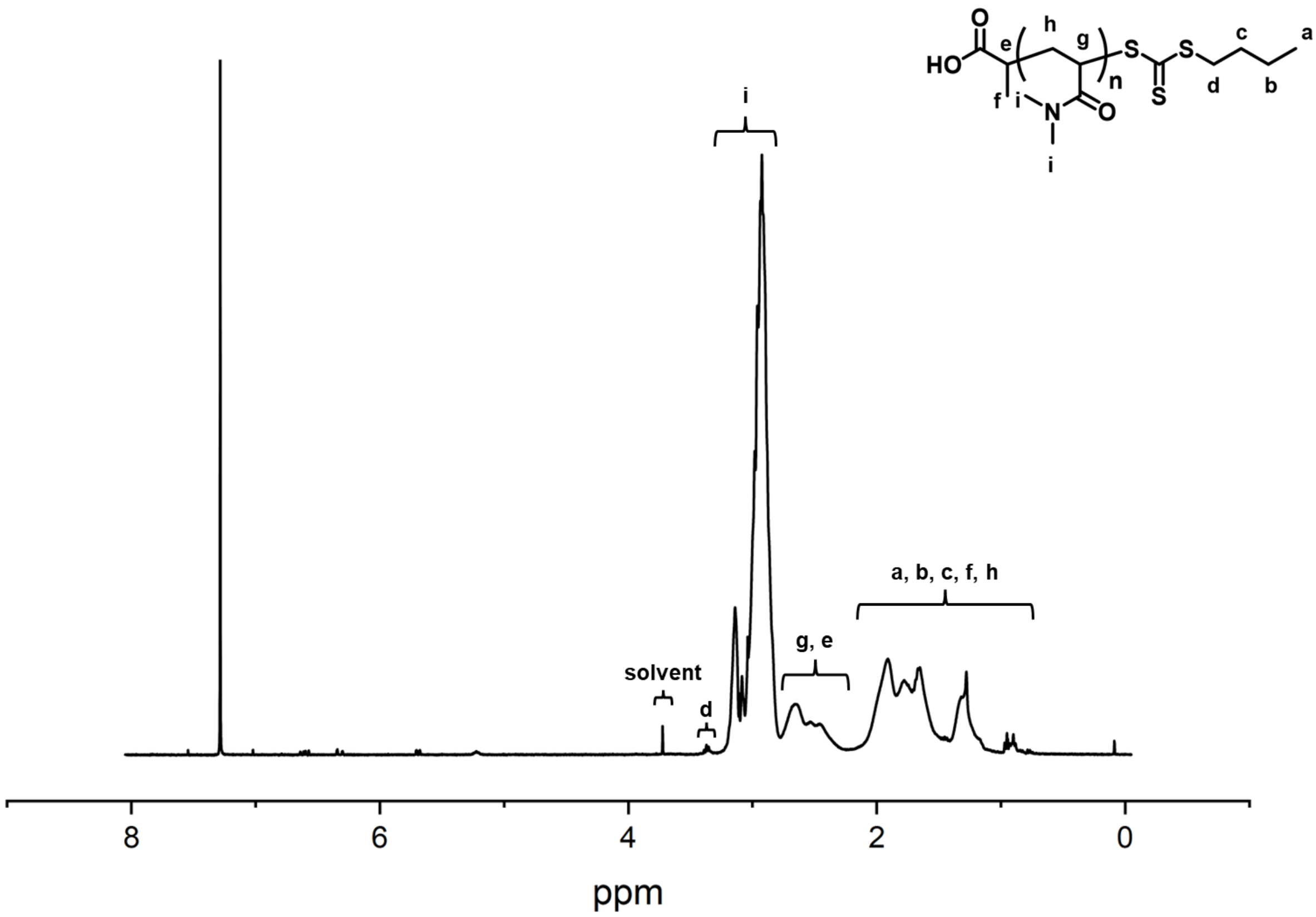

Figure S5. Assigned ^{1}H NMR spectrum for the $PDMAm_{75}$ macro-CTA used in this work

Automated experiment results

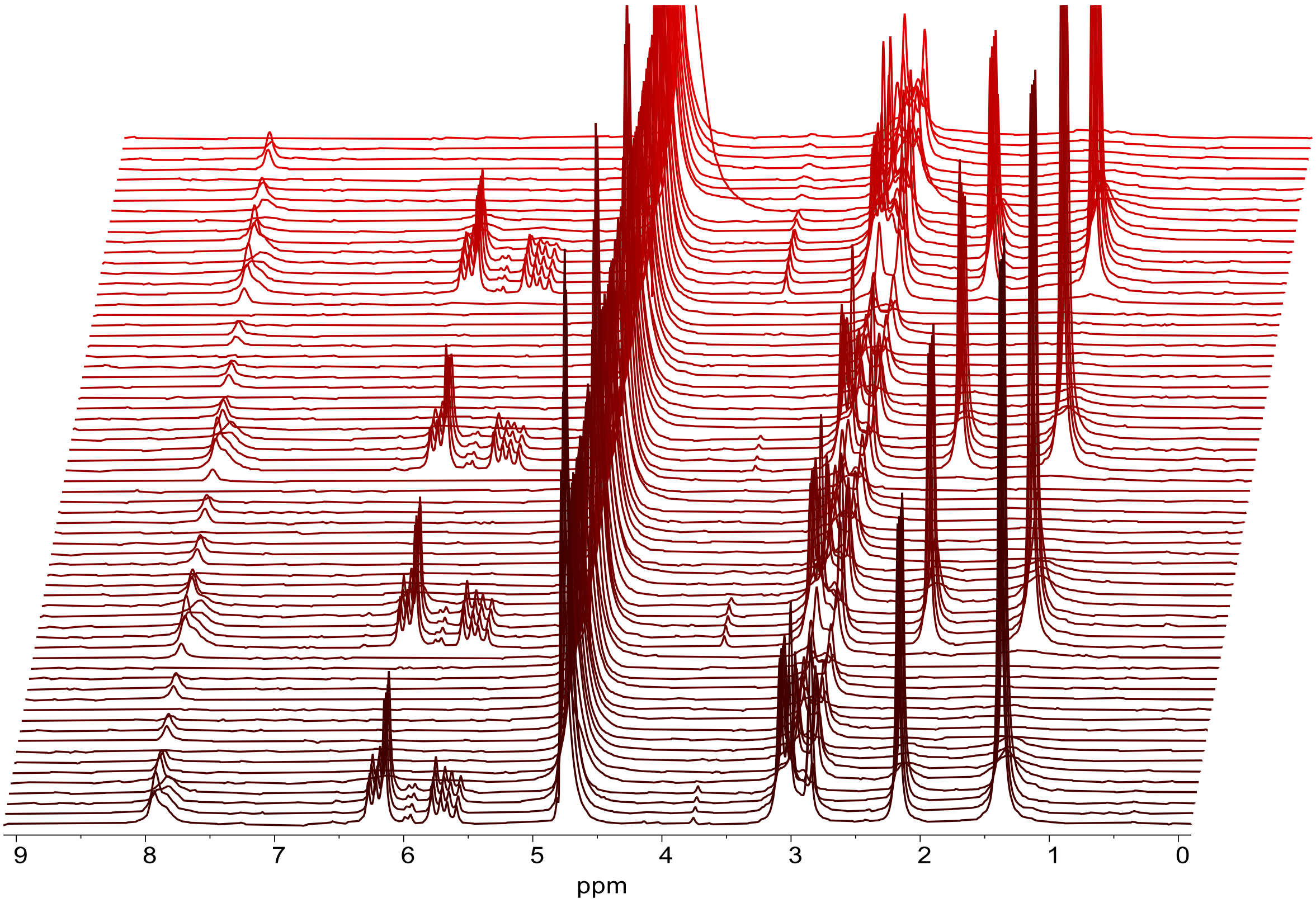

Figure S6. ^{1}H NMR spectra for the 4 x 4 x 4 screen for the RAFT dispersion polymerisation of DAAm in pH 2.5 water, using $PDMA_{74}$ as the macro-chain transfer agent and VA-044 as the initiator.

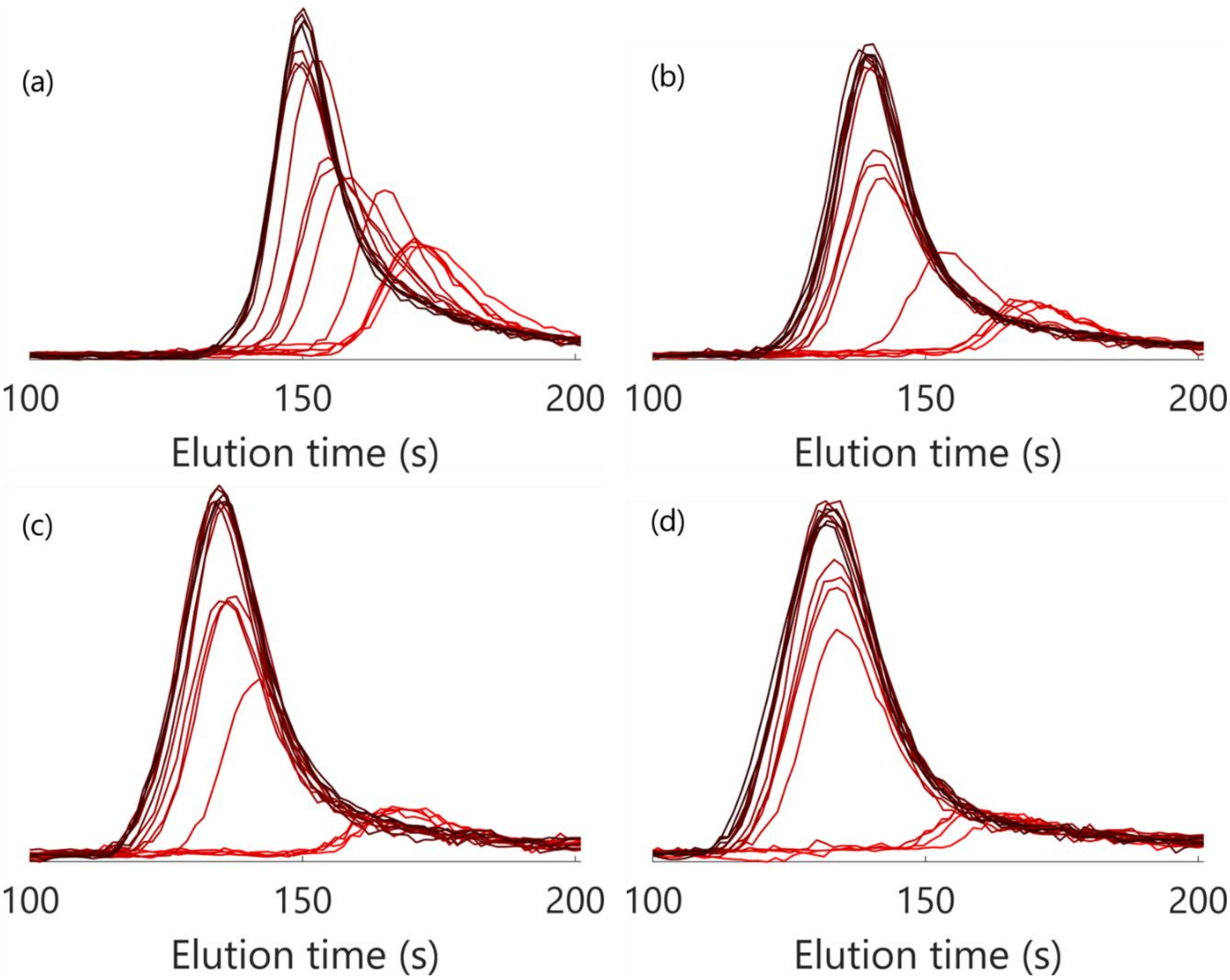

Figure S7. Raw chromatograms from GPC in the 4 x 4 x 4 screen for the four levels of [M]:[CTA]: (a) 100, (b) 267, (c) 433 and (d) 600 for the RAFT dispersion polymerisation of DAAm in pH 2.5 water, using $PDMA_{74}$ as the macro-chain transfer agent and VA-044 as the initiator.

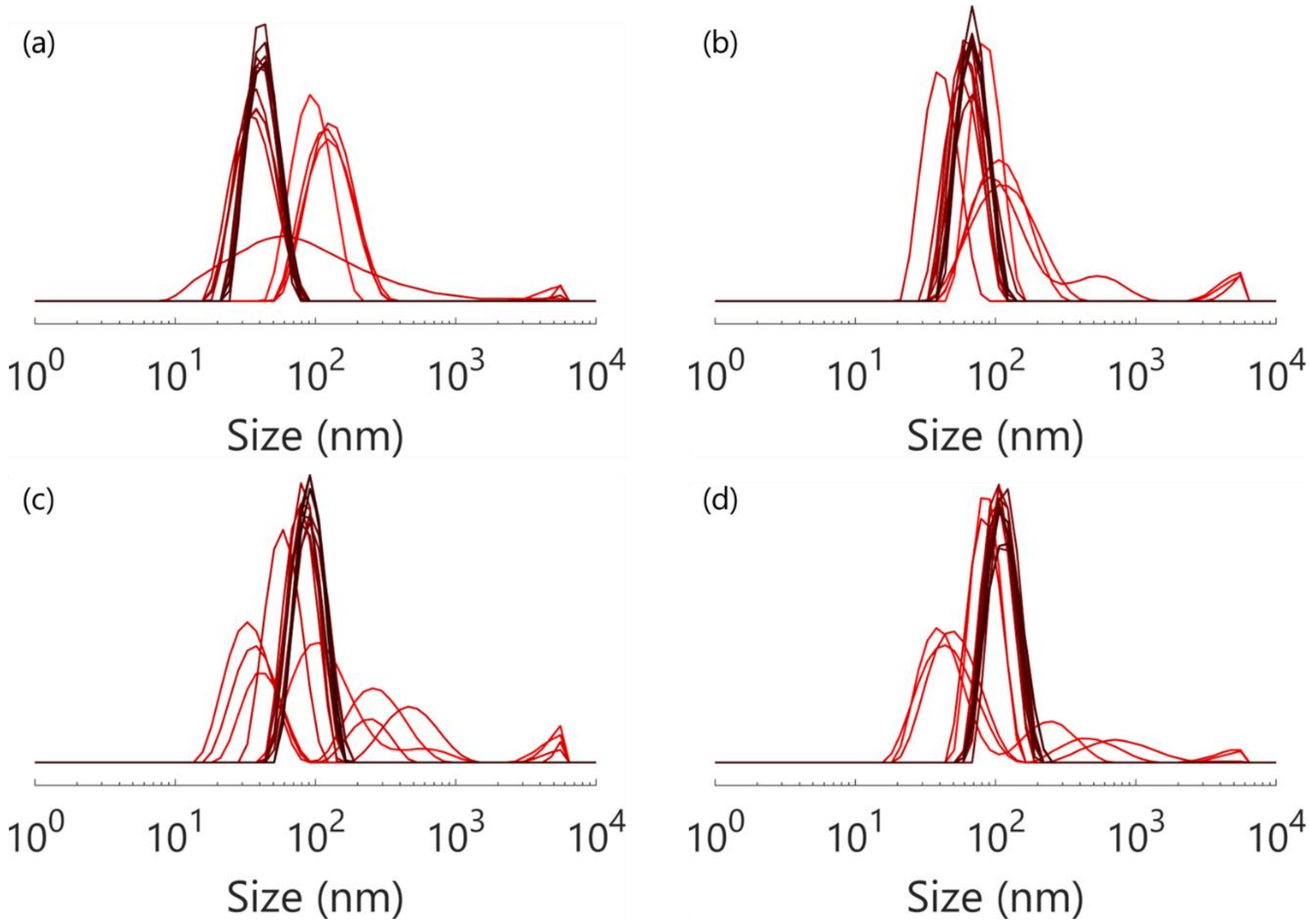

Figure S8. Intensity particle size distributions from DLS in the 4 x 4 x 4 screen for the four levels of [M]:[CTA]: (a) 100, (b) 267, (c) 433 and (d) 600 for the RAFT dispersion polymerisation of DAAm in pH 2.5 water, using $PDMA_{74}$ as the macro-chain transfer agent and VA-044 as the initiator.

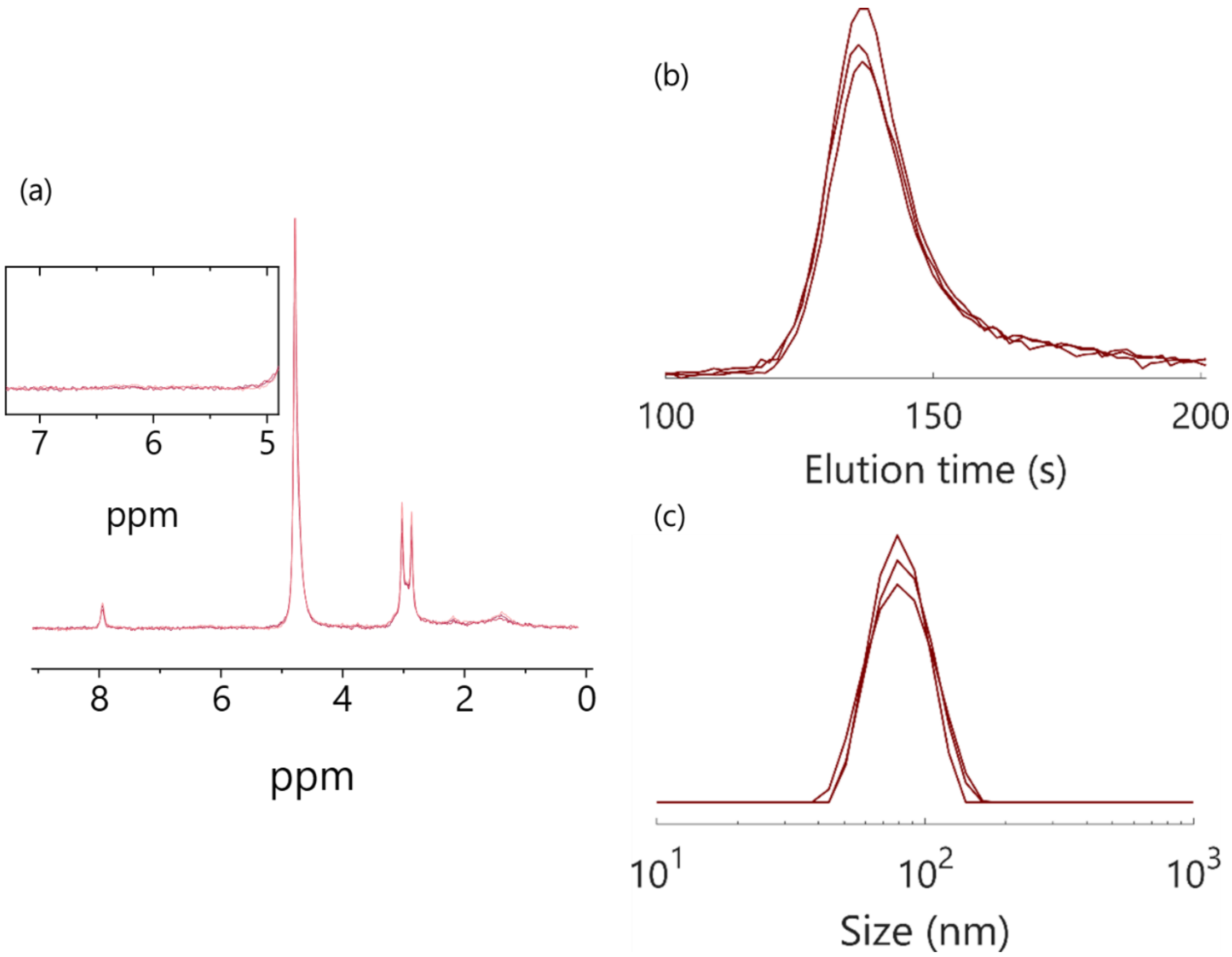

Figure S9. (a) ^{1}H NMR spectra, (b) Raw GPC chromatograms and (c) DLS intensity particle size distributions for the three repeats at the centre point (17.5 mins, 74 °C, [M]:[CTA] = 350) of the 4 x 4 x 4 full factorial screen for the RAFT dispersion polymerisation of DAAm in pH 2.5 water, using $PDMA_{74}$ as the macro-chain transfer agent and VA-044 as the initiator

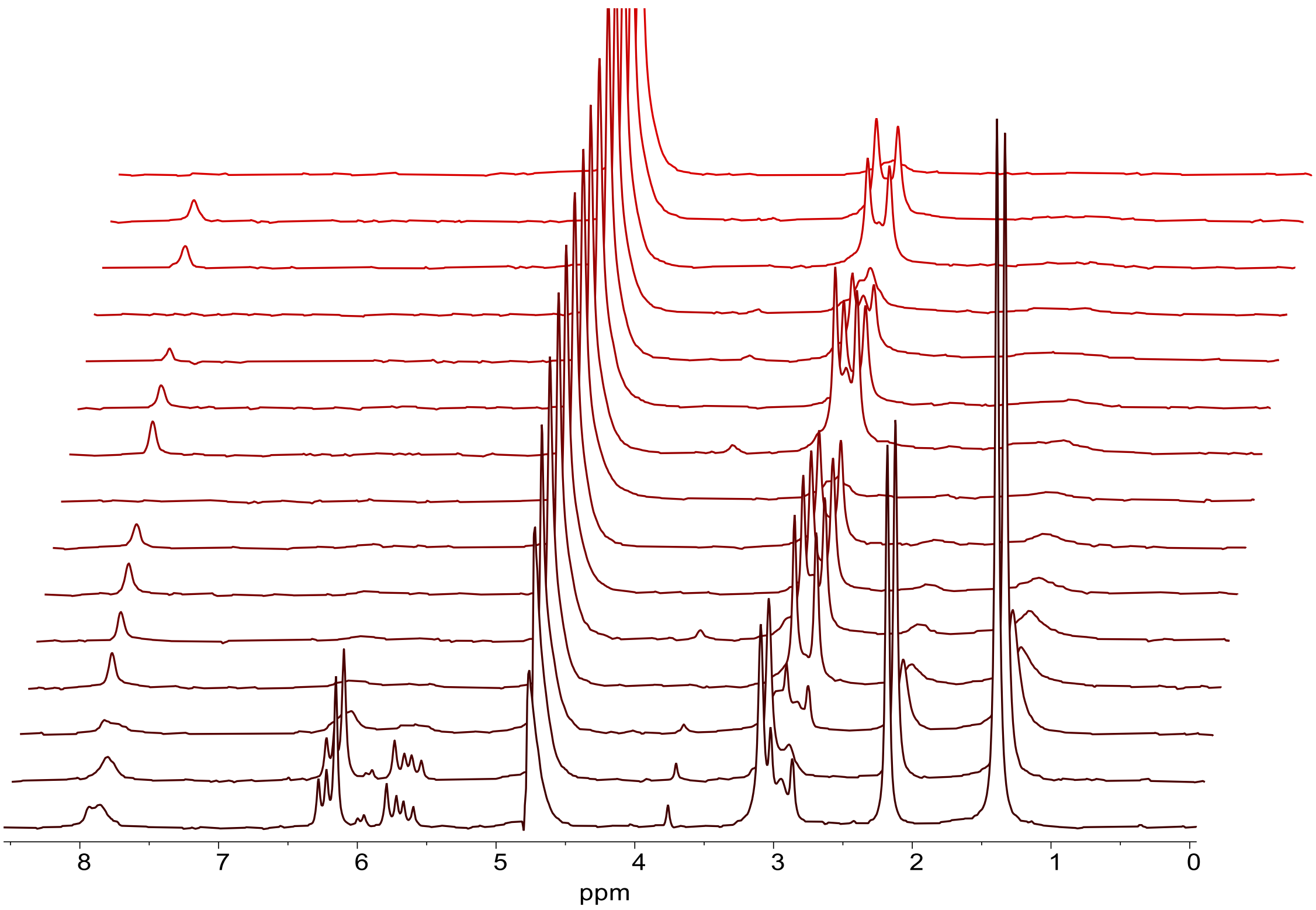

Figure S10. NMR spectra for the 15 experiments selected using Latin Hypercube sampling, for the RAFT dispersion polymerisation of DAAm in pH 2.5 water, using $PDMA_{74}$ as the macro-chain transfer agent and VA-044 as the initiator.

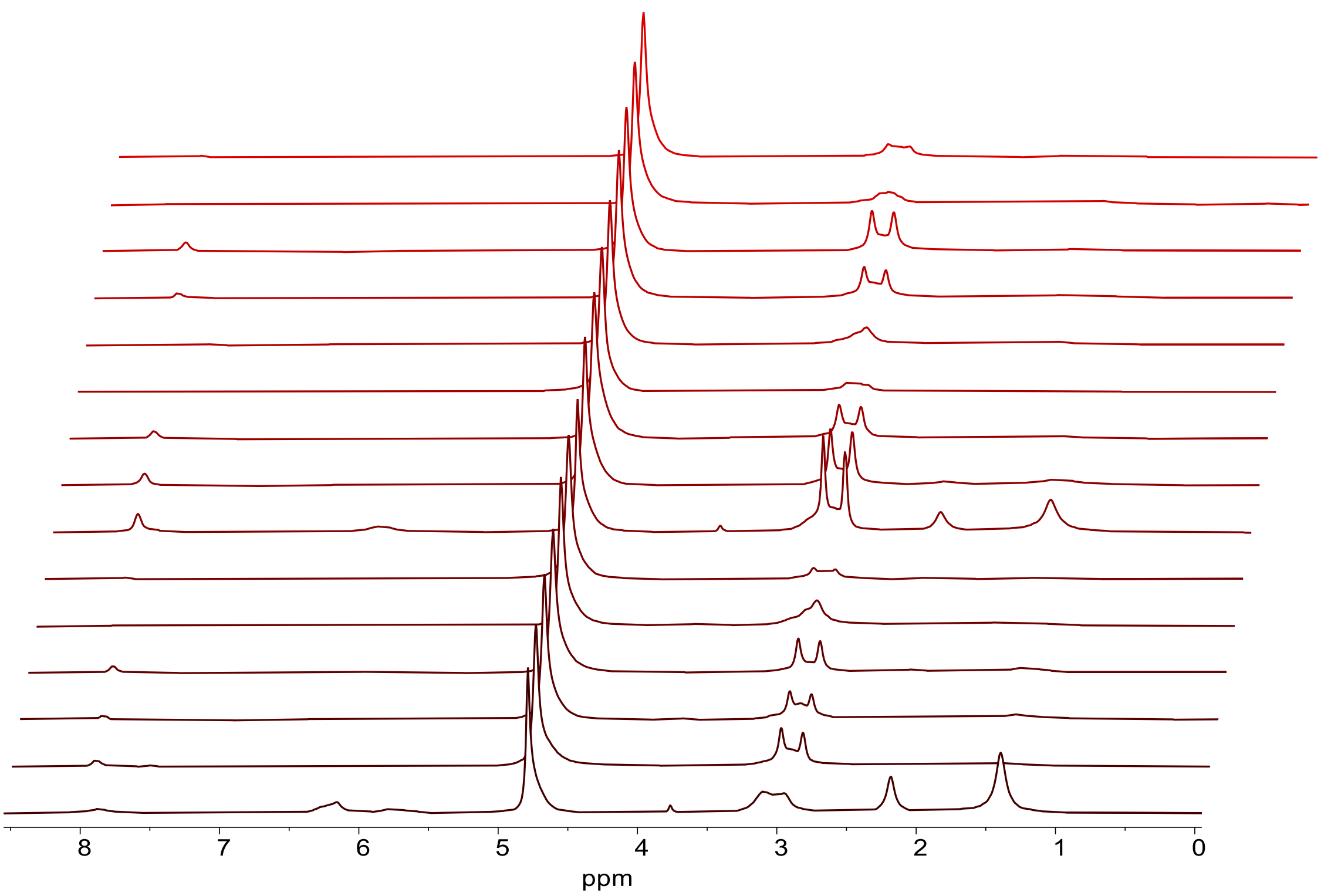

Figure S11. NMR spectra for the 15 experiments selected using the TSEMO algorithm, for the RAFT dispersion polymerisation of DAAm in pH 2.5 water, using $PDMA_{74}$ as the macro-chain transfer agent and VA-044 as the initiator.

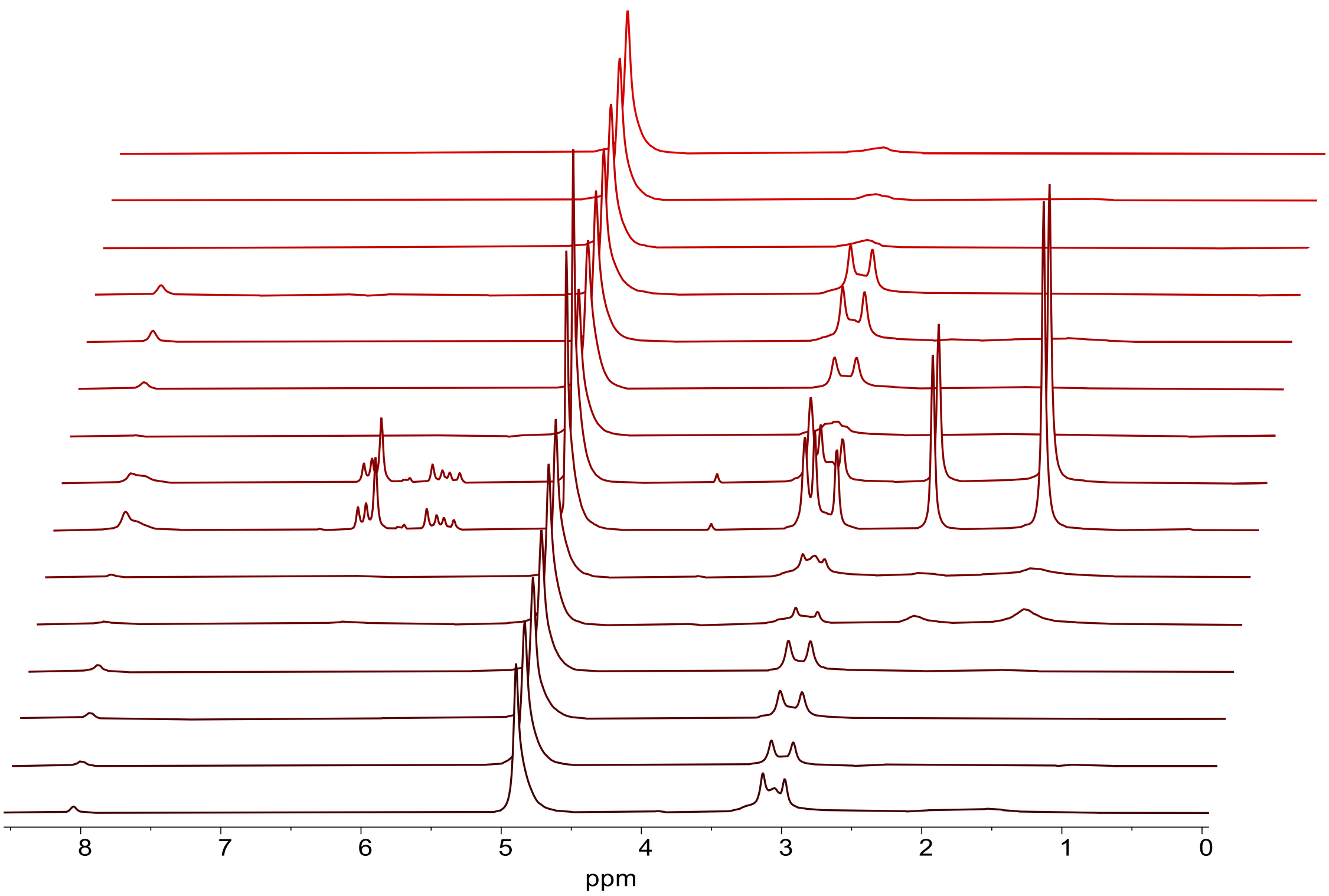

Figure S12. NMR spectra for the 15 experiments selected using the RBFNN/RVEA algorithm, for the RAFT dispersion polymerisation of DAAm in pH 2.5 water, using $PDMA_{74}$ as the macro-chain transfer agent and VA-044 as the initiator.

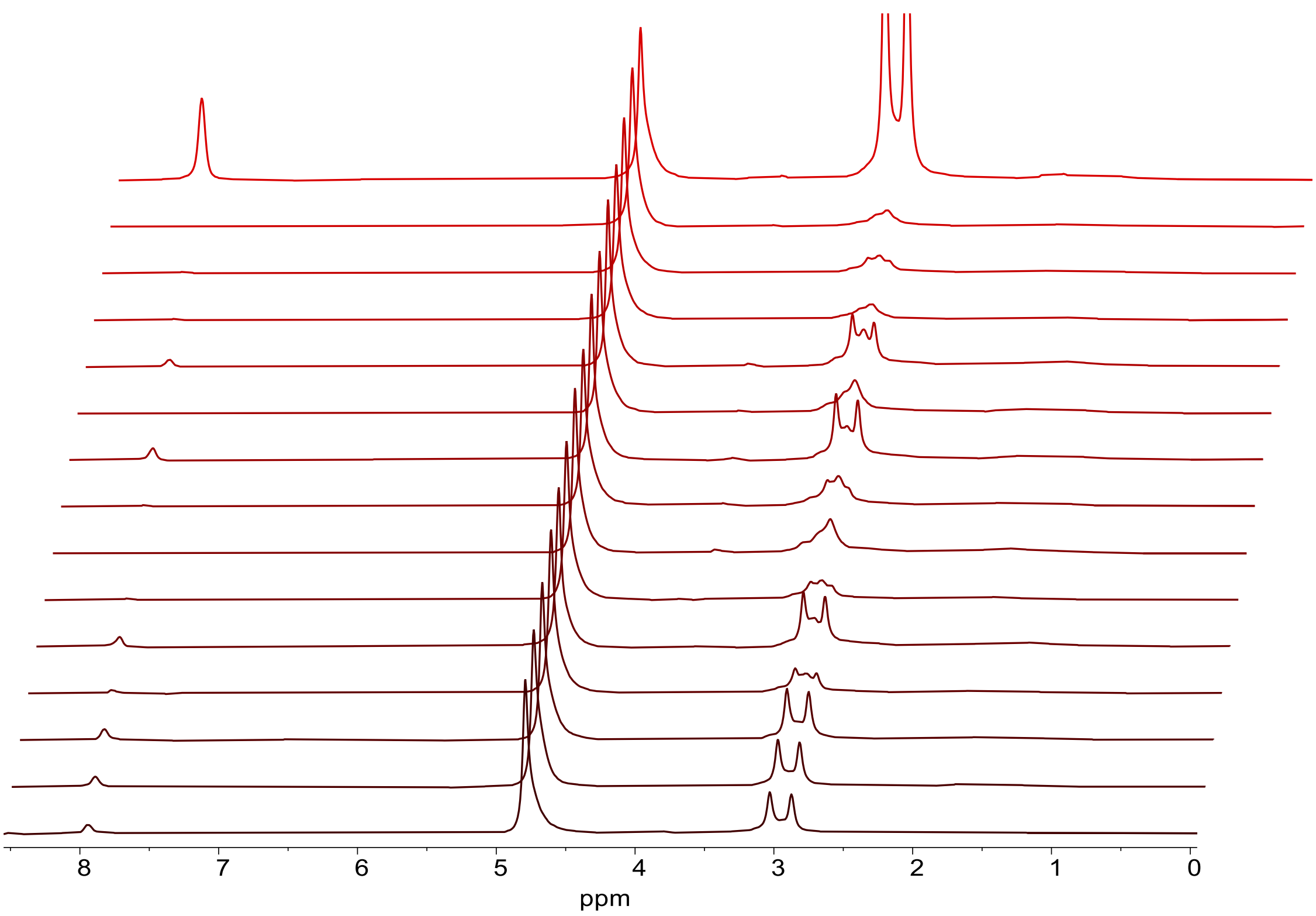

Figure S13. NMR spectra for the 15 experiments selected using the MOPSO algorithm, for the RAFT dispersion polymerisation of DAAm in pH 2.5 water, using $PDMA_{74}$ as the macro-chain transfer agent and VA-044 as the initiator.

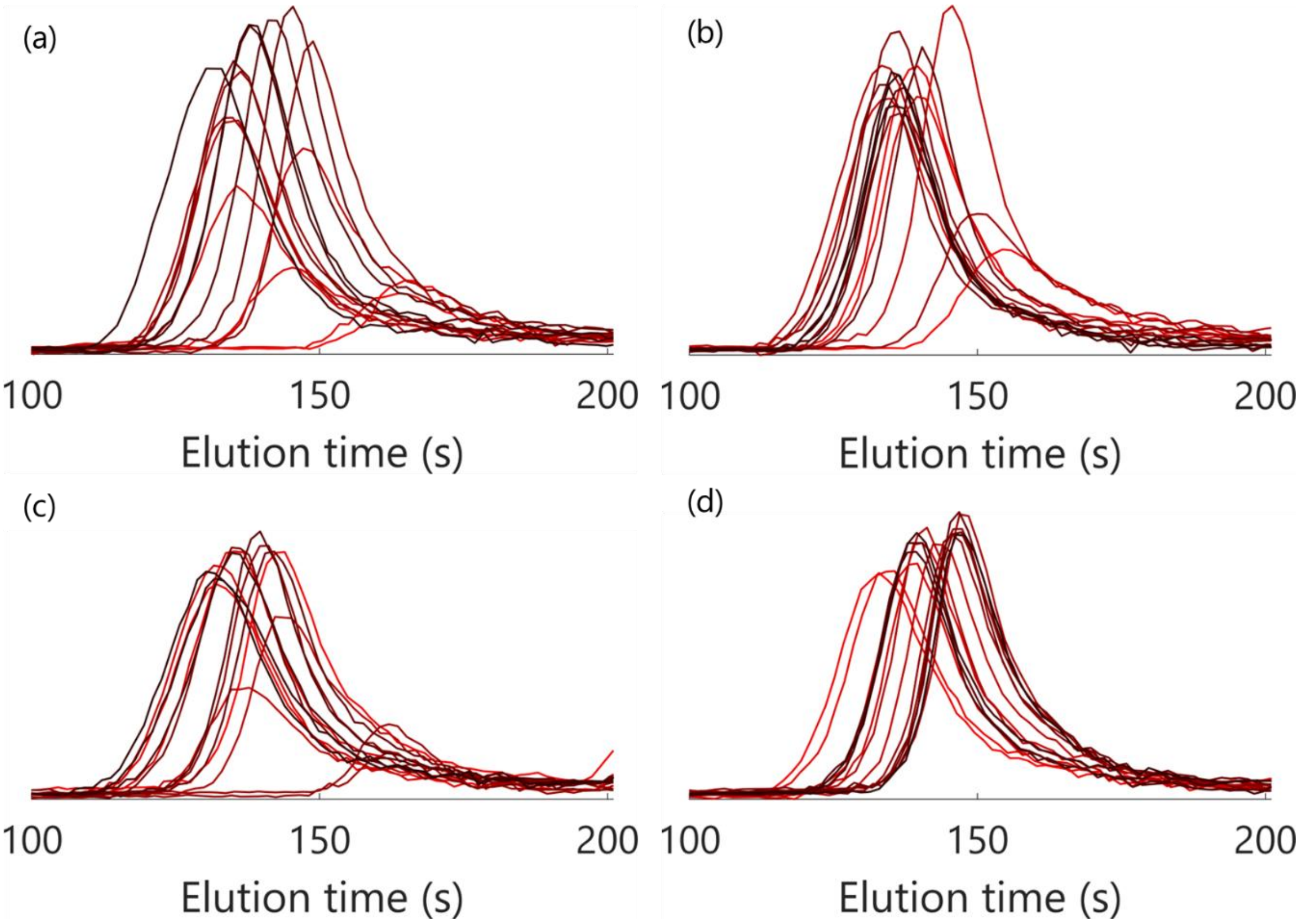

Figure S14. GPC chromatograms for (a) the 15 experiments selected using Latin Hypercube sampling, (b) the 15 experiments selected using the TSEMO algorithm, (c) the 15 experiments selected using the RBFNN/RVEA algorithm, and (d) the 15 experiments selected using the MOPSO algorithm; for the RAFT dispersion polymerisation of DAAm in pH 2.5 water, using $PDMA_{74}$ as the macro-chain transfer agent and VA-044 as the initiator.

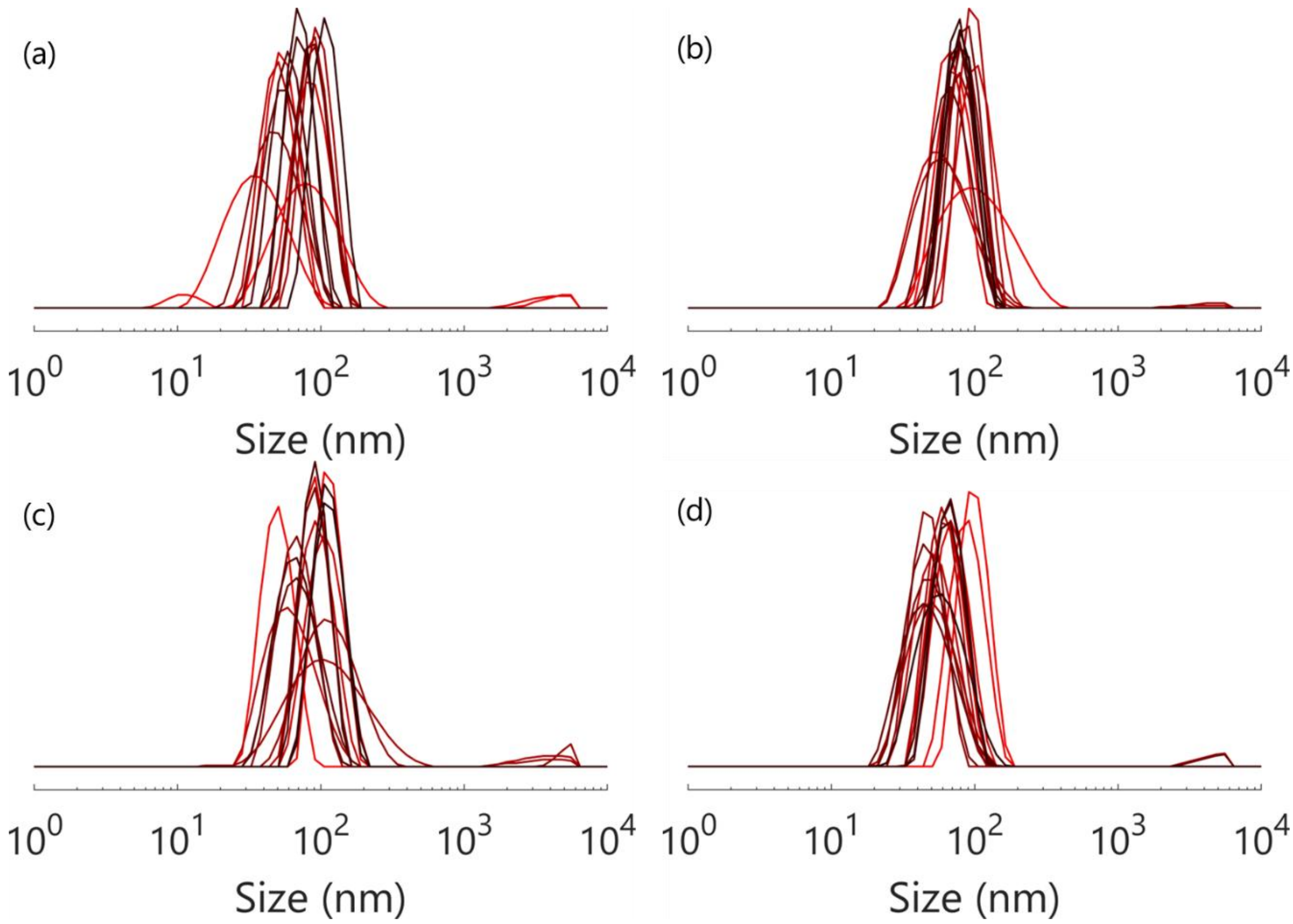

Figure S15. Intensity particle size distributions for (a) the 15 experiments selected using Latin Hypercube sampling, (b) the 15 experiments selected using the TSEMO algorithm, (c) the 15 experiments selected using the RBFNN/RVEA algorithm, and (d) the 15 experiments selected using the MOPSO algorithm; for the RAFT dispersion polymerisation of DAAm in pH 2.5 water, using $PDMA_{74}$ as the macro-chain transfer agent and VA-044 as the initiator.

Table S2. Each of the experiments conducted as part of the high throughput screen (4x4x4 – three **centre-point datapoints shown in bold iterations 17,34,51**), the Latin hypercube sampling (LHS) and the three optimisation campaigns (TSEMO, RVEA, MOPSO)

Exp	Iteration	RT (mins)	T (°C)	[M]:[CTA]	Conversion (%)	Dispersity	M_n (g mol^{-1})	M_p (g mol^{-1})	Particle size (nm)	PDI
4x4x4	1	6	68	433	1.99	1.43	6279	8497	114.3	0.47
4x4x4	2	14	68	433	3.11	1.94	6804	11484	80.0	0.75
4x4x4	3	22	68	433	0.38	1.53	7329	13459	52.7	0.29
4x4x4	4	30	68	433	8.28	2.47	7200	11513	53.0	0.64
4x4x4	5	6	72	433	74.95	1.83	61275	113784	56.7	0.06
4x4x4	6	14	72	433	92.84	1.87	108879	185965	76.8	0.01
4x4x4	7	22	72	433	90.78	1.81	109873	177078	80.5	0.01
4x4x4	8	30	72	433	93.16	1.88	116666	206145	83.1	0.00
4x4x4	9	6	76	433	99.06	1.81	142176	219062	80.4	0.04
4x4x4	10	14	76	433	99.91	1.95	145827	236508	89.2	0.04
4x4x4	11	22	76	433	99.56	2.00	135494	227031	92.3	0.02
4x4x4	12	30	76	433	96.11	1.83	153265	231361	90.7	0.02
4x4x4	13	6	80	433	98.46	1.85	148100	232987	82.6	0.04
4x4x4	14	14	80	433	99.73	2.02	164719	262680	89.6	0.03
4x4x4	15	22	80	433	95.31	2.05	141227	220779	90.9	0.04
4x4x4	16	30	80	433	96.38	2.14	149743	254185	91.3	0.01
4x4x4	18	6	68	267	3.44	1.81	6138	7914	82.3	0.02
4x4x4	19	14	68	267	4.62	1.30	7004	10919	108.3	0.30
4x4x4	20	22	68	267	8.30	1.34	6706	10428	114.2	0.35
4x4x4	21	30	68	267	0.80	1.31	6938	11103	112.4	0.53
4x4x4	22	6	72	267	47.80	1.55	18239	30422	39.0	0.08
4x4x4	23	14	72	267	79.04	1.82	50773	96553	56.2	0.04
4x4x4	24	22	72	267	89.51	1.73	59843	104041	58.1	0.05

4x4x4	25	30	72	267	92.10	1.75	64032	110079	60.0	0.05
4x4x4	26	6	76	267	98.74	1.55	86594	124116	62.3	0.01
4x4x4	27	14	76	267	99.99	1.63	86621	142833	66.3	0.07
4x4x4	28	22	76	267	98.90	1.65	91597	145715	68.1	0.00
4x4x4	29	30	76	267	97.70	1.60	96880	145487	68.6	0.01
4x4x4	30	6	80	267	99.99	1.52	92746	129777	64.9	0.04
4x4x4	31	14	80	267	99.26	1.63	100815	159315	66.6	0.02
4x4x4	32	22	80	267	99.99	1.62	91897	134200	68.1	0.02
4x4x4	33	30	80	267	95.17	1.63	96501	142911	67.8	0.03
4x4x4	35	6	68	600	8.08	2.33	7404	12060	84.0	0.01
4x4x4	36	14	68	600	14.26	2.36	8431	18955	53.2	0.36
4x4x4	37	22	68	600	13.84	1.82	8085	21271	53.8	0.36
4x4x4	38	30	68	600	16.33	2.61	8163	23826	57.4	0.34
4x4x4	39	6	72	600	86.70	1.98	130691	227329	81.2	0.04
4x4x4	40	14	72	600	92.88	2.20	151668	304847	99.7	0.02
4x4x4	41	22	72	600	93.09	2.21	161848	268579	105.4	0.08
4x4x4	42	30	72	600	95.00	2.23	170844	320845	108.2	0.00
4x4x4	43	6	76	600	96.34	2.07	185180	304593	101.1	0.02
4x4x4	44	14	76	600	99.99	2.22	186258	373441	110.1	0.04
4x4x4	45	22	76	600	97.00	2.17	189299	373651	109.6	0.04
4x4x4	46	30	76	600	97.39	2.09	194311	347496	116.3	0.00
4x4x4	47	6	80	600	99.74	2.15	180530	320901	103.2	0.03
4x4x4	48	14	80	600	98.57	2.19	180881	380693	106.0	0.04
4x4x4	49	22	80	600	95.19	2.20	182126	351544	109.3	0.03
4x4x4	50	30	80	600	97.28	2.25	176241	320775	111.8	0.05
4x4x4	52	6	68	100	2.49	1.18	6211	8435	89.1	0.08
4x4x4	53	14	68	100	2.34	1.17	6788	9118	122.1	0.25
4x4x4	54	22	68	100	6.29	1.19	6986	8777	112.4	0.17
4x4x4	55	30	68	100	5.71	1.19	7216	9591	123.4	0.34
4x4x4	56	6	72	100	21.11	1.15	10586	13795	52.8	0.46

4x4x4	57	14	72	100	63.72	1.34	14122	22586	34.4	0.15
4x4x4	58	22	72	100	68.01	1.38	16753	27476	36.0	0.09
4x4x4	59	30	72	100	69.37	1.42	16816	28567	36.3	0.09
4x4x4	60	6	76	100	94.21	1.28	25783	34269	35.7	0.07
4x4x4	61	14	76	100	99.99	1.38	30297	45417	39.4	0.06
4x4x4	62	22	76	100	96.43	1.37	31003	47959	40.6	0.04
4x4x4	63	30	76	100	96.07	1.37	30759	46333	40.8	0.05
4x4x4	64	6	80	100	99.99	1.29	34979	47435	38.7	0.06
4x4x4	65	14	80	100	99.99	1.28	33124	45404	41.1	0.02
4x4x4	66	22	80	100	97.84	1.31	33815	43611	41.2	0.02
4x4x4	67	30	80	100	96.04	1.30	33061	44654	41.4	0.06
**4x4x4**	**17**	**18**	**74**	**350**	**98.55**	**1.73**	**116848**	**191458**	**78.0**	**0.06**
**4x4x4**	**34**	**18**	**74**	**350**	**97.80**	**1.74**	**122028**	**194594**	**78.5**	**0.00**
**4x4x4**	**51**	**18**	**74**	**350**	**94.83**	**1.79**	**108392**	**177987**	**79.8**	**0.04**
LHS	1	21	69	258	8.92	1.28	8378	12546	67.9	0.30
LHS	2	28	69	358	11.90	1.62	7957	16422	34.5	0.26
LHS	3	22	70	443	52.00	2.51	23768	76525	51.9	0.04
LHS	4	16	71	570	80.41	2.16	106428	228369	82.1	0.05
LHS	5	18	72	169	86.42	1.72	30231	59601	48.6	0.04
LHS	6	8	73	516	94.30	1.94	154767	243785	86.4	0.02
LHS	7	15	73	495	92.90	2.13	137601	240381	93.4	0.01
LHS	8	28	74	392	99.99	1.88	134445	224357	85.7	0.07
LHS	9	11	75	115	99.79	1.44	33075	48515	46.5	0.13
LHS	10	10	75	405	99.99	1.81	136801	221406	84.5	0.02
LHS	11	20	77	142	99.99	1.40	49755	67620	53.3	0.07
LHS	12	25	77	206	99.99	1.50	71369	109696	58.1	0.05
LHS	13	13	78	292	97.53	1.63	102730	161273	70.0	0.02
LHS	14	7	79	301	98.89	1.61	106536	160282	69.6	0.01
LHS	15	27	80	562	95.25	2.11	200567	386715	106.6	0.01
TSEMO	1	27	70	275	52.34	1.66	14706	28554	90.5	0.19

TSEMO	2	14	74	370	97.71	1.68	86250	138703	70.9	0.03
TSEMO	3	22	73	253	95.22	1.61	82413	132890	63.9	0.05
TSEMO	4	21	73	539	97.35	2.05	174329	292408	98.5	0.05
TSEMO	5	28	78	135	99.86	1.33	50577	69075	55.6	0.15
TSEMO	6	23	79	482	98.42	2.18	165424	276985	96.4	0.03
TSEMO	7	11	71	221	65.27	1.84	18872	41087	59.0	0.18
TSEMO	8	15	73	336	92.04	1.74	115330	184752	75.4	0.09
TSEMO	9	18	78	357	99.99	1.70	146338	196692	81.5	0.04
TSEMO	10	25	78	375	93.24	1.73	192450	293838	85.3	0.04
TSEMO	11	30	79	201	97.78	1.38	89741	119204	64.7	0.07
TSEMO	12	20	75	302	99.19	1.52	132615	191775	74.4	0.02
TSEMO	13	9	78	333	99.53	1.66	144279	225239	77.5	0.04
TSEMO	14	27	75	296	99.18	1.59	145216	203884	76.2	0.02
TSEMO	15	23	78	327	98.27	1.58	153084	205093	78.9	0.01
RVEA	1	20	76	150	97.08	1.62	50028	80783	48.5	0.04
RVEA	2	20	76	572	96.84	2.05	189633	344792	111.0	0.00
RVEA	3	6	80	449	98.12	2.13	140443	247615	88.8	0.02
RVEA	4	7	79	598	98.94	2.08	186961	363153	103.2	0.06
RVEA	5	30	71	573	75.23	2.58	67793	169071	89.1	0.09
RVEA	6	24	73	173	89.90	1.70	38768	84304	61.7	0.22
RVEA	7	7	68	368	6.67	1.32	10915	16327	100.1	0.28
RVEA	8	20	69	234	11.27	1.22	12723	15925	112.2	0.31
RVEA	9	27	76	232	95.98	1.63	86593	133055	64.9	0.07
RVEA	10	18	78	418	99.99	1.89	151052	265560	87.7	0.04
RVEA	11	15	75	208	97.97	1.60	67989	119892	65.5	0.13
RVEA	12	16	76	225	99.99	1.63	79266	135157	62.8	0.08
RVEA	13	30	80	408	93.83	2.18	144055	245049	89.8	0.00
RVEA	14	28	79	548	94.31	2.27	166513	397245	108.6	0.05
RVEA	15	30	80	544	93.74	2.20	186005	350318	110.5	0.02
MOPSO	1	7	76	498	99.41	2.09	170528	298254	96.5	0.00

MOPSO	2	11	75	399	99.13	1.98	140023	235133	85.7	0.04
MOPSO	3	7	75	293	99.84	1.63	98097	154764	67.0	0.05
MOPSO	4	22	77	205	99.53	1.56	73467	117474	61.6	0.04
MOPSO	5	18	76	145	98.41	1.54	52358	94494	50.3	0.10
MOPSO	6	24	79	199	99.99	1.51	74937	115056	58.8	0.03
MOPSO	7	30	80	100	99.99	1.38	37505	61012	50.5	0.21
MOPSO	8	24	77	119	99.99	1.42	44832	68642	45.8	0.03
MOPSO	9	17	77	117	99.00	1.43	40854	63460	45.9	0.11
MOPSO	10	27	78	107	99.83	1.37	41119	59464	43.5	0.07
MOPSO	11	19	78	100	99.99	1.35	39074	60660	46.1	0.20
MOPSO	12	28	79	243	99.99	1.61	100857	140384	64.8	0.04
MOPSO	13	25	76	225	97.58	1.59	83960	131626	62.9	0.07
MOPSO	14	27	77	234	95.19	1.59	87877	137929	65.5	0.03
MOPSO	15	19	77	100	94.83	1.38	37688	63806	55.2	0.11

References

1 S. T. Knox, S. J. Parkinson, C. Y. P. Wilding, R. A. Bourne and N. J. Warren, *Polym. Chem.*, 2022, **13**, 1576–1585.

2 E. Bradford, A. M. Schweidtmann and A. Lapkin, TS-EMO, https://github.com/Eric-Bradford/TS-EMO.

3 A. M. Schweidtmann, A. D. Clayton, N. Holmes, E. Bradford, R. A. Bourne and A. A. Lapkin, *Chem. Eng. J.*, 2018, **352**, 277–282.

4 Y. Tian, R. Cheng, X. Zhang and Y. Jin, *IEEE Comput. Intell. Mag.*, 2017, **12**, 73–87.

5 N. Islam and J. Oyekan, in *Conference on Genetic and Evolutionary Computation*, Lisbon, 2023.
